# Development of a (digital) mindfulness-informed intervention for older adults in nursing homes: description and reflection of a person-based co-design approach

**DOI:** 10.1186/s12877-025-06223-x

**Published:** 2025-09-25

**Authors:** Maren M. Michaelsen, Jule Uhl, Luka Mindrup, Claudia Neumann, Lena Langer, Tobias Esch

**Affiliations:** Institute for Integrative Health Care and Health Promotion, Faculty of Health, Witten/Herdecke University, Alfred-Herrhausen-Str. 50, Witten, 58448 Germany

**Keywords:** Person-based co-design, Nursing homes, Mindfulness, Digital mindfulness, MHealth, Intervention development, Participatory research, Mind–body medicine

## Abstract

**Background:**

Mindfulness and Positive Psychology interventions have proven effective in enhancing psychosocial well-being and cognitive abilities among older adults residing in long-term care facilities. However, the implementation of health promotion measures in this population remains limited. Participatory intervention development with this target group is rare and processes of generating intervention content, format, and delivery as well as design decisions are often not well-documented. Our research aim was to use person-based co-design (PBCD) methods to facilitate the translation of meaningful stakeholder experiences into the design of an on-site and digitally delivered mindfulness-informed intervention (MII) for health promotion in nursing homes.

**Methods:**

The PBCD process involved multiple stakeholders, including the target group, their relatives, nurses and other experts from nursing homes, health insurance representatives and app developers. The iterative process comprised several key steps: theory-based selection of mind–body exercises by the project team, informal discussions with intervention experts, semi-structured interviews with the target audience (*n* = 3) during 12 sessions, pre-test of the exercises through an online survey with researchers (*n* = 15), online survey with project partners, nursing facility experts, caregivers, and the general public (*n* = 12). Qualitative feedback was assessed through deductive-inductive content-structuring analysis. For the digital version, the app developers created two front-end application examples, of which one was further refined based on regular feedback from the target audience and the research team.

**Results:**

MII (named silBERN) for health promotion comprising preparatory elements (welcoming, general information), 22 exercises and 13 exercise repetitions in 8 modules with a delivery plan over 8 weeks. 11 exercises were adopted and 8 exercises were excluded based on feedback from different stakeholders. User experience was incorporated in the app version. The main feedback referred to the complexity/comprehension of exercises. A resource-oriented view of the target group was implemented. The PBCD process proved valuable despite its time-intensive nature.

**Conclusion:**

Stakeholder participation has led to interventions that fit the needs of the target group. The process of engaging all stakeholders in the PBCD process can be time-consuming and intensive. Careful reflection on the development process indicates that a preliminary phase of participation was carried out. A randomized controlled trial of the silBERN intervention is required to evaluate usability and effectiveness.

**Supplementary Information:**

The online version contains supplementary material available at 10.1186/s12877-025-06223-x.

## Background

### Introduction

Cognitive impairments as well as depression are prevalent among older individuals residing in long-term care facilities [1–3]. However, the implementation of intervention measures aimed at enhancing psychosocial well-being and cognitive abilities in this population within residential care settings remains limited [4]. The German “Prevention in Long-Term Care Facilities” guideline highlights the dearth of evidence regarding the effectiveness of interventions to strengthen these aspects in care facilities [5].

Thus, a four-year collaborative project was conducted with the primary objective of promoting cognitive performance and psychosocial health in older adults residing in nursing homes through the implementation of a mindfulness-informed health promotion intervention to be delivered either in a group setting with a trainer (analogue) or digitally on an individual basis with technical support (digital).

### Mindfulness

Mindfulness is a cognitive state and meditative practice rooted in various contemplative traditions, including Buddhism, where individuals purposefully direct their attention to the present moment’s sensory experiences and mental phenomena without judgment, fostering self-awareness and mental clarity [6]. It involves cultivating an open and receptive attitude, acknowledging thoughts and emotions as they arise [7], and has been widely incorporated into secular therapeutic interventions to promote psychological well-being, stress reduction, and emotional regulation [8, 9]. Mindfulness practice typically centers on anchoring attention to the breath, bodily sensations, or other sensory inputs, enhancing metacognitive awareness [10] and potentially facilitating positive cognitive and emotional outcomes. The taught exercises within mindfulness-enhancing programs can typically be described as either (a mindfulness-based exercises and procedures that develop mindfulness directly and (b mindfulness-informed exercises that promote mindfulness through an understanding of the connection and mutual influence of body and mind [11, 12]). In many interventions, mindfulness is practiced in order to enhance well-being and quality of life, a goal shared by exercises rooted in Positive Psychology [13]. Often, these exercises focus on aspects related to gratitude, forgiveness, optimism, savouring, curiosity, courage, altruism and meaning of life, which have been shown to be effective among healthy older adults [14].

One of the most widespread programs to promote mindfulness is Mindfulness-based Stress Reduction (MBSR). MBSR is a program developed by Jon Kabat-Zinn that integrates mindfulness meditation and other mind–body practices in an eight-week program offered in a group format. The program intends to help individuals manage stress by cultivating present-moment awareness, enhancing emotional regulation, and fostering a healthier relationship with thoughts and emotions, initially developed to help patients suffering from pain [15]. It has been researched extensively [12, 16–18] and is utilized to complement medical and psychological treatments, showing positive effects on psychological well-being and physical health in many different (patient) populations [19].

For older adults, MBSR programs appear similarly effective. A number of studies were conducted with individuals aged 65 and older living in their own homes, with some populations feeling lonely or being depressive. The authors found positive effects on a large array of outcomes, including perceived stress [20], awareness, self-reflection and self-acceptance [21], mood [22], relaxation and interpersonal connection [23], immune function [24, 25], as well as cognitive function [26]. A systematic review [27] found mixed results for memory, executive function and processing speed, however, and a meta-analysis on six studies provided mixed results for stress and anxiety, but positive effects on depression [28]. For older adults living in a continuing care retirement community, Moss et al. [29] implemented a mixed-methods approach to analyze the impact of MBSR training and found significantly greater improvement in acceptance, psychological flexibility and in role limitations in the 80 + year-old intervention group compared to a randomized control group. Ernst et al. [30] show that a satisfactory number of participants aged 72–98 years can complete the MBSR course (60% in the feasibility study). In their study, participants show significant improvement in the SF-12 physical health summative scale, a decrease in depressive symptoms, and there is a potential to achieve improvements in mental health and life satisfaction.

In addition to the widely known MBSR program, a mindfulness-based program adapted to the abilities and needs of older individuals has been developed, named Mindfulness-Based Elder Care (MBEC) [31]. The effectiveness of MBEC has been evaluated, for example, in Hsiung et al. [32], who conducted a randomized controlled trial (RCT) among seniors with disabilities in long-term care residential settings and found improvements in anxiety, depressive symptoms and spiritual well-being. MBSR and related formats have been approved generally feasible in older adults [33], however, studies conducted for institutionalized older adults remain extremely limited. Building on the preliminary insights from a feasibility study involving the implementation of a MBSR course in a German nursing home [30] and the insights provided by McBee [31] and Hsiung et al. [32], in this study, a participatory approach to develop a mindfulness-informed intervention (MII) for this specific target group is described and reflected.

### Health promotion course BERN

Scientifically evaluated programs in the field of mindfulness interventions, such as the above described MBCR and MBEC programs, are typically rooted in the topics of cognitive **b**ehavior, **e**xercise, **r**elaxation, and **n**utrition – the four pillars of the BERN concept [34, 35]. The elements of a health promotion course based on these principles, called BERN course, clearly show beneficial effects on participants with different chronic diseases [35]. In the University Outpatient Clinic for Integrative Health Care and Health Promotion of Witten/Herdecke University, a multiprofessional primary health care institution, the BERN course is provided for patients with diverse indications in eight modules in a group setting on-site, since December 2020 and also as an online group course during the Covid-19 pandemic. The themes of each module include stress and stress management, relaxation, mindful movement, nutrition, social network, language and expression, self-help and self-healing, as well as relapse prevention (see Table 1).

**Table 1:** Theory- and evidence-based intervention draft comprised of 26 exercises in 8 modules

Module	Exercise	Description
Module 1: Stress and Stress Management	Setting Goals	Defining personal goals as part of stress management
	Source of Strength	Identifying personal resources and supportive memories
	New and Good	Focusing attention on positive experiences or events
Module 2: Relaxation	Diaphragmatic Breathing	Breathing technique to activate the relaxation response
	Fist Exercise	Progressive muscle relaxation using hand tension and release
	Meditation – Relaxation Response	Meditation to activate the body’s natural relaxation state
	Meditation in Daily Life	Integrating meditation as a regular part of the daily routine
	Minis	Short relaxation exercises for everyday use
Module 3: Mindfule Movement	Body Scan	Guided practice to build body awareness
	Yoga	Gentle mindful movement with attention to one's physical limits
	Qi Gong – Meridians	Gentle energy-based movements, including work with meridians
	Qi Gong – Regulate the Breath	Breathing and movement exercise for regulation and relaxation
	Walking Meditation	Meditative walking practice with awareness of the steps
	Movement Prescription	Personalized plan or encouragement for regular movement
	SARW – Be Mindful	Awareness practice with focus on attention and acceptance
Module 4: Nutrition	Mindful Eating Exercise (Raisin Exercise)	Mindful awareness while eating a small item like a raisin
	Mediterranean Diet – Mindful Drinking	Practice mindfulness during drinking, connected to Mediterranean diet principles
	20 Things → 3 Things (Treasure Chest)	Reducing and focusing on a few meaningful items or practices
	Guided Imagination “Kitchen”	Visualization related to a kitchen environment to explore feelings and associations
Module 5: Social Network	Social Atom – Relationship Network	Mapping and reflecting on personal social connections
Module 6: Language and Expression	Postcard	Creative expression through writing or drawing on a postcard
	Loving-Kindness Meditation	Meditation focused on sending goodwill and compassion to oneself and others
Module 7: Self-Help and Self-Healing	Meaning and Purpose (Self-Help Strategies)	Exploring personal meaning and self-care approaches
	Jokes/Humor/Laughter	Encouraging laughter and humor for emotional well-being
Module 8: Relapse Prevention	Prescription – Self-Care Health Plan	Creating a personalized plan for long-term well-being
	Conclusion	Summary and overview of practices, encouragement to continue independently

### Theory- and evidence-base for the present intervention

For the purpose of our study, content of the MBSR and BERN programs was selected and adapted to the specific target group of residents in nursing homes. This serves as a theory- and evidence base for the intervention. In addition to MBSR and BERN protocols, existing experience from the MBEC model [31] were considered. One of the main difference between MBEC compared to MBSR and BERN protocols is that it was developed for an older population, in which the significance of goals is not as prominent as it is for younger individuals, who are generally the target groups of the other protocols. According to Lucia McBee [31], in the MBEC framework, the focus for seniors is more on letting go of goals. At the same time, “letting go” is also a key outcome of mindfulness meditation. Using a participatory approach, the exercises were further developed according to the needs and resources of the target group.

### Participatory intervention development

Traditionally, intervention development guidelines primarily focus on constructing a solid theoretical foundation for a one-size-fits-all intervention and then conducting experimental tests to validate the assumed causal relationships [36, 37]. However, they tend to neglect practical aspects related to the intervention’s implementation and feasibility until later stages [38, 39]. As a result, even if an intervention is theoretically well-founded and supported by evidence, it may fail to be effective or adopted by the target group due to various translational factors [40–42]. For example, patients or healthcare providers might not perceive the intervention as useful or relevant to their needs. Additionally, if the intervention is difficult to navigate or implement within the existing healthcare system, it may face resistance or challenges in gaining widespread acceptance [43].

Therefore, there is currently a strong emphasis on involving patients and the public in health research [44]. The emphasized participatory approaches aim to address the aforementioned shortcomings by actively involving stakeholders throughout the intervention development process [37, 45]. These individuals become active partners in the research process, sharing power and influence over its various stages and outcomes [46–48]. By including stakeholders early on, their perspectives, preferences, and concerns can be considered from the outset, ensuring that the intervention is not only theoretically sound but also practically acceptable and feasible [46–48].

The foundation of participatory research rests on two essential principles: inclusivity and democracy. These principles foster effective cooperation and power-sharing among stakeholders, particularly those directly affected by the research [46]. Inclusivity ensures that individuals, who are the focus of the research, actively participate in the process, while democracy ensures they have equal influence and decision-making power alongside research professionals. These collaborative approaches increase the likelihood of successful implementation and uptake of the intervention, ultimately leading to improved healthcare outcomes [46].

However, it is not fully understood how to execute this engagement effectively and meaningfully to achieve genuinely patient-centered research [44]. Therefore, several methodologies have been proposed, such as community-based participatory research [49], action research (e.g., [50]), co-design (e.g., [51]), person-based approaches [52], intervention mapping (e.g., [53]), user-centered design (e.g., [54, 55], and others, which align with these principles. These methodologies share the common goal of involving stakeholders and use specific protocols and methods.

As our target group is older adults in long-term care facilities with cognitive impairments, we adopted principles from these approaches that suited the setting and intention of the study in the best way possible. The approach being employed in this study can be described as a person-based co-design (PBCD) approach.

The person-based approach originates from the field of health psychology and serves as a framework for crafting interventions setting out to foster health-related behavioral changes, whether involving digital components or not [52, 55]. We believe this approach is crucial for determining the most suitable and effective intervention design elements tailored to our specific population. Qualitative research plays a central role in the person-based approach throughout all stages of intervention development and assessment, spanning planning, design, initial development, acceptability and feasibility testing, as well as evaluation in clinical trials and real-world scenarios [55].

In addition to a person-based approach, co-design represents a methodology that not only accumulates knowledge across successive development cycles [56] but also places special emphasis on integrating diverse stakeholders, reconciling conflicting requirements, and swiftly transitioning concepts into testable prototypes. Like other design approaches, co-design employs a variety of tools and exercises to facilitate collaboration between professional designers and individuals typically untrained in design processes, such as patients and therapists [51]. When executed effectively, co-design amalgamates varied perspectives, inputs, and skill sets to address specific issues [57]. Consequently, this approach enhances the acceptability and integration of interventions into existing clinical practice by accommodating pertinent contextual factors identified by stakeholders in the developmental process.

Based on these principles, we allowed for the integration of perspectives and preferences from those who will ultimately benefit from the research in order to create an analogue group MII and a digital individual MII that are patient-centered and contextually appropriate.

### Objectives

The study aimed to explore the application of a PBCD approach in developing an analogue (on-site) group MII called *silBERN* that is subsequently translated for individual use into a digital format (app) for older individuals in nursing homes. By actively involving stakeholders, the research sought to collect knowledge and experiences, prioritize objectives, and generate solutions. The main objectives were to understand how PBCD practices translate meaningful stakeholder experiences into intervention design based on pre-defined principles (foundational framework BERN, salutogenic approach, feasibility) and to provide an overview and reflection of the PBCD activities employed in this project.

## Methods

### Target group of the intervention

The study was registered in the German Register of Clinical Studies (ID: DRKS00030409). Ethics vote (IRB) was obtained from the ethics committee of the University Witten/Herdecke (no. 233/2020). The planned MII planned to include residents of inpatient care facilities (nursing homes) in Germany who voluntarily agree to participate. Inclusion criteria have been outlined together with care facility employees, requiring participants to fulfill specific criteria, including age (≥ 70 years), cognitive capability (Minimal Mental Status Test—MMST ≥ 20), and the capacity to actively engage in group activities, participate in group discussions, communicate effectively, and comprehend and execute exercise instructions. Informed consent to participate was obtained from all of the participants in the study.

### Planned delivery framework of interventions

Based on these prerequisites, we allowed for the integration of perspectives and preferences from those who will ultimately benefit from the research in order to create an intervention (on-site and app-versions) that is patient-centered and setting-based. For the goal of testing the intervention in a randomized controlled trial [58], it was planned to establish study groups each comprising 5–7 nursing home residents receiving i) group exercise sessions under the guidance of certified BERN trainers (on-site), ii) engagement with an app-assisted exercise program (app), or iii) control condition, i.e., adhering to the facility’s customary program (treatment as usual) throughout the intervention phase.

The group exercise sessions, led by a trainer (qualified therapist for health promotion; experienced with the target group), were planned to be scheduled to take place once a week (50–60 min), while the app-based intervention was intended as a form of an outreaching health promotion measure entailing for example two shorter sessions per week (20 min each session) in the residents’ room. In the app-based intervention, residents would be guided one-on-one by assistants to engage in mindfulness-informed exercises facilitated through the app. These assistants may encompass caregivers, volunteers, relatives, and other participants, who will undergo preparatory training and receive a comprehensive manual to proficiently administer and accompany the exercises informed by prior insights and feedback. It was planned that a tablet would serve as the primary medium for conducting the app-supported exercises. Notably, the exercises themselves, whether presented within the on-site sessions or through the app-supported intervention, were tried to maintain uniformity and consistency.

### Stakeholder involvement

The initial project outline developed in 2019 envisioned a participatory intervention development workshop with 20 participants from various stakeholder groups. The start of the project coincided with the beginnings of the COVID-19 pandemic and the resulting restrictions on face-to-face meetings. Therefore, these workshops could not be held. Instead, alternatives were developed to enable participatory principles.

The project’s person-based co-design approach entailed the active involvement of a diverse range of stakeholders. These stakeholders include participants and individuals directly impacted by the project, such as those from the target demographic and their family members. Additionally, experts from nursing facilities, i.e. individuals involved in caregiving, regardless of their specific training, contributed their insights. This includes professionals such as nursing practitioners holding licenses, as well as other caregiving roles like nursing assistants, care aides, and other caregiving personnel within the facilities. Additionally, researchers from different disciplines and teams, positive psychology intervention experts, as well as general citizens were included in the project.

The overall project was accompanied by a project advisory board. Members of the advisory board represent the fields of health and nursing science, the business sector (operators of care facilities), funding entities (represented by a delegate from the project funder), as well as a political representative and family members of residents in a care facility. Additionally, nursing professionals and/or nursing home managers are included in the project advisory board. It was decided to appoint the funder of this project (association of statutory health insurance funds) in the role of the representative of the health insurance sector. With one representative per outlined role, the advisory board consisted of seven members.

### Intervention development steps

**Fig. 1:**
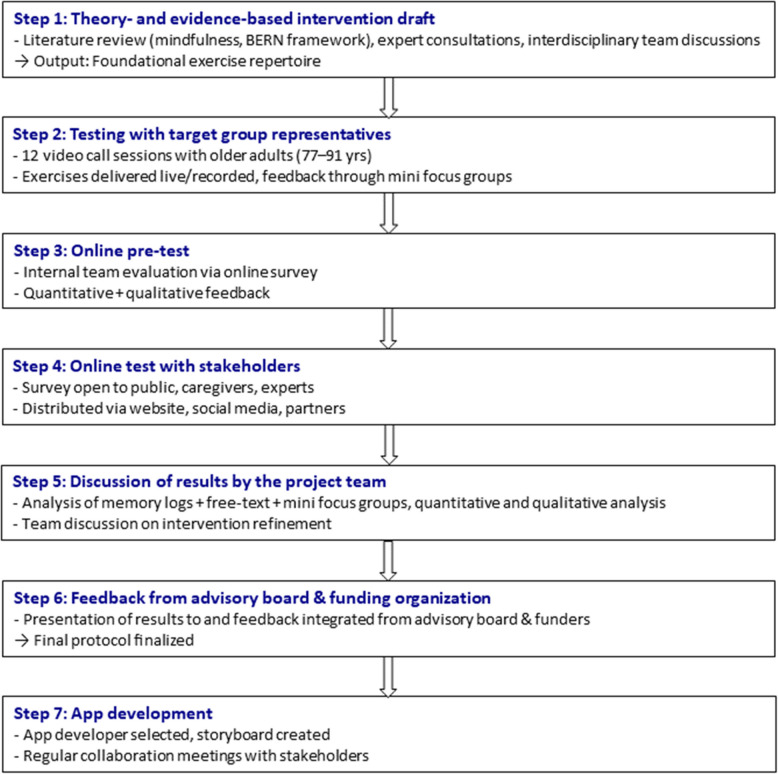
Flowchart of the seven-step intervention development process. The process began with a literature- and theory-informed intervention draft (Step 1), followed by participatory testing and validation involving the target group and stakeholders (Steps 2–6). Finally, the intervention protocol was translated into a digital format through structured app development (Step 7)

All in all, seven steps involving person-based and co-design methods were pursued. All participants were comprehensively informed about the project in advance (content, procedure, type of exercises) in accordance with the guidelines for patient participation [59]. A role definition was created, and stakeholders were specifically invited to participate as representatives of the relevant roles. This inclusive stakeholder collaboration aimed to ensure a comprehensive and multifaceted perspective throughout the project’s development and implementation. The seven steps are illustrated in Fig. 1 and described more detailed in the following.

#### Step 1: theory- and evidence-based intervention draft

**Fig. 2:**
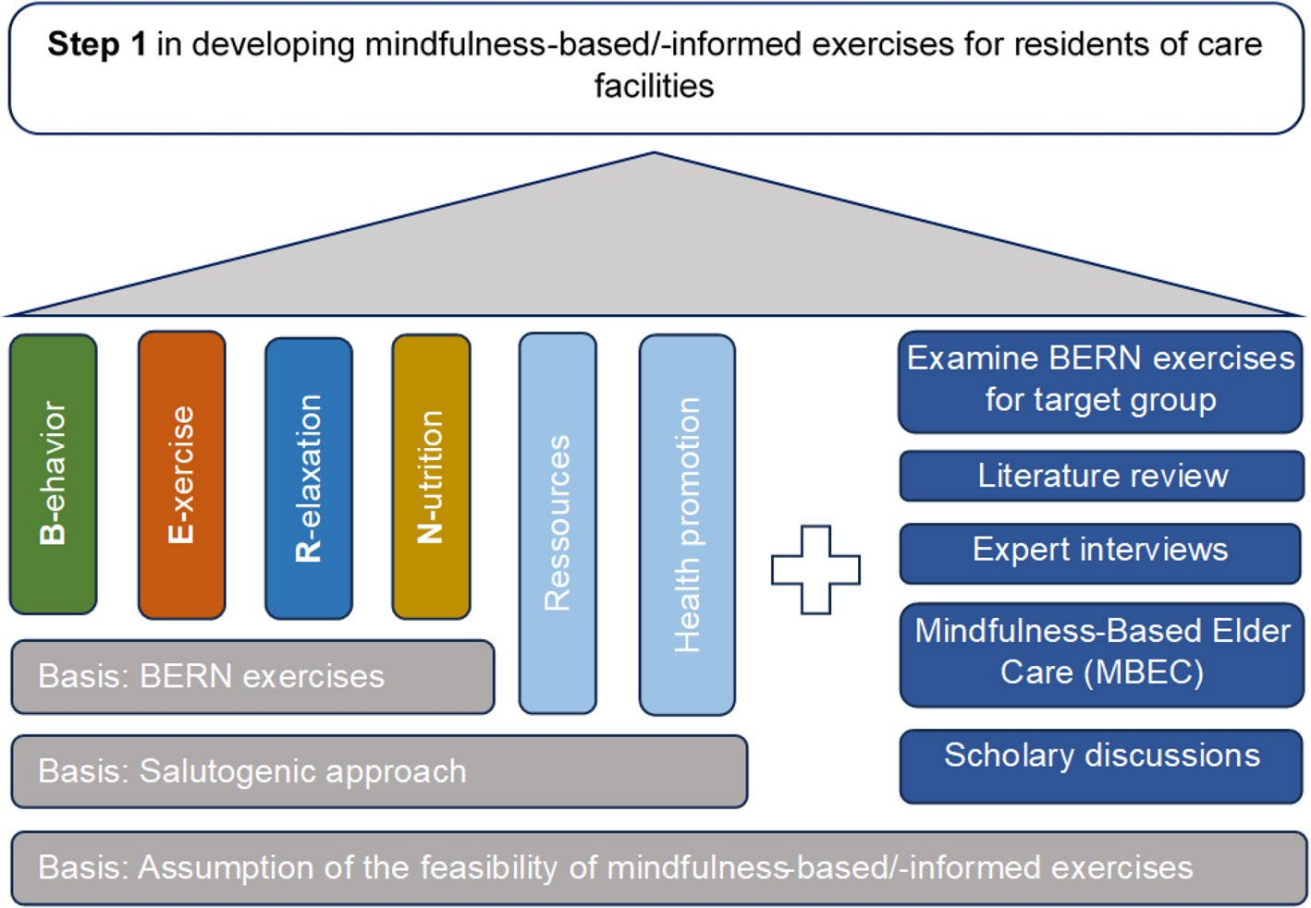
Intervention development components—Part 1

The planning process integrated theory and empirical findings with the collection and utilization of new data to formulate interventions [60]. As a first step of the process (described in Fig. 2), a comprehensive literature review was conducted, focusing on the utilization of mindfulness-based and -informed exercises within the study’s specific target population. As a foundation for exercise development, the BERN health promotion exercises were adopted, alongside an inherent salutogenic approach in implementation and the underlying assumption of the feasibility of mindfulness-based and -informed exercises within the targeted study demographic, as demonstrated in Ernst et al. [30]. Furthermore, insights derived from the MBEC program [31] were incorporated into this process.

This was followed by two expert interviews with positive psychology intervention practitioners who teach the approach “*Humor Hilft Heilen* (humor supports healing)” in clinical settings. Based on the insights generated through the study of the literature, the BERN framework was adopted by a subset of the author team. The outcomes of these evaluations were subject to thorough discussions among researchers of the Institute of Integrative Health Care and Health Promotion (IGVF), including a content expert interview with the head of the institute, a discussion with the advisory board, a scholarly discussion group with researchers spanning various fields (including psychology, public health, behavioral economics, medicine, neuroscience, nursing) within the Faculty of Health at the University of Witten/Herdecke. Some of these researchers also have experience in nursing and/or are yoga and meditation teachers besides their research activities.

Collectively, this endeavor resulted in the establishment of a foundational repertoire of 26 exercises – the theory- and evidence-based intervention draft – serving as the basis for subsequent iterative refinements through participatory methodologies (see Table 1).

#### Step 2: testing with target group representatives

The exercises selected and adapted were carried out for an initial test in 12 online video call sessions of 1.5 h involving three representatives of the target group and then discussed in detail with them afterwards (mini focus groups). These subjects were in the age range of 77 to 91 years in the year of the survey, 2021, and were residents of a nursing facility that supports the project as a practice partner. They performed all exercises according to the instructions that were either delivered live and online by one of the researchers or by a video that was recorded previously by the research team. Based on semi-structured interview questions (see Appendix 1a), the representatives of the target group gave comprehensive feedback on the exercises and their experience of performing them. Finally, they evaluated the overall course. The sessions were recorded in form of memory protocols summarizing the results of the sessions.

#### Step 3: online pre-test

As a further element of the participatory development, all planned exercises were evaluated anonymously by researchers at the IGVF in a pre-test for the planned online survey. The survey contained quantitative and qualitative feedback questions about the delivery and content of each exercise presented by the same videos as in Step 2.

#### Step 4: online test with stakeholders

Based on the pre-test, the survey was slightly adopted for the online survey with a broader stakeholder audience. The exercises were distributed via the same format as in Step 3 via a four-week anonymous feedback survey to representatives of the project practice partners, to experts from care facilities and caregivers, and to the interested public via a web link on the IGVF homepage. The link was also distributed in social media and on the website of the association for caring relatives (www.wir-pflegen.nrw.de). At the beginning of the survey, the participants were asked (optionally) to which group of persons (role) they assigned themselves. From the answers, it can be seen whether and in what number persons of the defined roles took part. The surveys have been developed for this study and are provided in Appendix 2. They contain statements that could be answered “yes” (agreement with the statement in question) or left blank (“not-yes”), as well as open-ended questions regarding suitability, difficulties and advantages of the exercise. Data were analyzed according to a protocol as described in the section “Data analysis”.

#### Step 5: discussion of results by the project team

We followed a systematic approach to collecting and condensing qualitative feedback. Based on the different types of data, memory logs were analyzed to ensure alignment between free-text responses and the statements of the target groups during the mini focus groups. Expert input by the senior author of this study informs the final approach to handling feedback. We conduct frequency counts in the qualitative analysis to gauge the urgency of adjustments needed. We also considered the transferability of feedback to other exercises and determine action steps based on relevance and alignment with our assumptions. Based on the analysis of the data acquired in the online surveys and mini focus groups with the target population, a theory-, evidence- and person-based intervention draft was presented to and discussed by the whole team of the IGVF. The discussion was recorded by a meeting minutes protocol.

#### Step 6: feedback from advisory board & funding organization

In a next step, the results were presented to the advisory board and funding organization (health insurance representatives). After incorporating feedback from these agents, the final exercises protocol was finished.

#### Step 7: app development

Based on the final protocol, the development of a mobile app comprised a series of distinct but interconnected sub-stages. The initial phase of app development involves identifying and selecting proficient app developers with the required expertise. Developers’ competence is paramount as it directly influences the quality and functionality of the end product. Based on a carefully established protocol, potential app developers were contacted, and after a rigorous evaluation, one developer was chosen who demonstrated a comprehensive understanding of the project’s objectives and technological requirements. In a next step, a storyboard was developed by the research team. Representatives from the project team, app developers, and health insurance representatives convened regularly to ensure constant collaboration. These meetings served to review progress, address challenges, and align the development trajectory with the project’s goals.

### Data analysis

The collaborative approach yielded both quantitative and qualitative data for each proposed exercise. The data acquired from the mini focus groups (Step 2) was incorporated directly while the answers of the two online surveys (Steps 3 and 4) were analyzed systematically based on the following principles.

#### Quantitative feedback

Based on the online survey, we counted the “yes” replies as well as the number of responses in the “other” field. As soon as at least one participant ticked one of the statements that contained negative criticism, we analyzed the qualitative feedback provided in order to understand the cause of the potential problem regarding the exercise or implementation of the exercise.

#### Qualitative feedback

The qualitative component of the intervention development framework serves the purpose of identifying and addressing adaptive requirements that may have been overlooked in the initial stages and quantitative analysis. This qualitative aspect plays a crucial role in uncovering potential obstacles that could hinder the implementation and adoption of interventions [61].

The evaluation of open-field responses was conducted using a content-structuring analysis methodology [62]. This analytical approach involves a combination of deductive and inductive processes to create categories. According to Kuckartz, deductive-inductive category formation [62] initially uses a small number of main categories as a basis. These are usually derived from the research question or theory and are considered merely as a starting point or “search grid” [62]. In our case, feedback was expected from the beginning in terms of (1) content, (2) presentation, and (3) wording. The procedure according to Kuckartz [62] is characterized by several iteration loops that are carried out. In these loops, the answers relevant to the research questions are assigned to the main category system and (sub-)categories formed on the material. Categories are grouped and summarized according to content proximity [62]. Coding was done by one researcher and validated by a second researcher of the research team.

### Framework for incorporating feedback

The subsequent framework delineates the procedure devised to assimilate both quantitative and qualitative feedback derived from the online surveys, focus groups, discussions and interviews with stakeholders. In order to facilitate this incorporation, three presumptions have been outlined for the further evolution of the intervention.

1. Maintenance of original project approach: BERN course integration.

The foundational framework as originally concurred upon with the project sponsor was intended to be maintained the basis of the intervention. The conceptual framework hinges on the BERN course, signifying a target group-specific adaptation of the course. In adherence to this framework, the project seeked to deepen insights into the potential benefits of mindfulness-based or -informed practices within the specified demographic. It is essential to highlight that this approach caters to target group characteristics and organizational specifics.

(2) Embracing a salutogenic approach.

The foundational underpinning of the planned study is embedded within a salutogenic ethos firmly entrenched in the domain of Integrative Medicine [11]. Salutogenesis is understood as “a paradigm that focuses on health (as opposed to disease) determinants and the development of individual resilience and coherence factors as a means to reduce stress, decrease the burden of disease, and improve the quality of life” [35, 63].

(3) Feasibility as a guiding premise.

An overarching principle of feasibility is established as a key consideration, stating that mindfulness-based and -informed exercises can indeed be effectively delivered within the intended target group. The implementation of this principle is based on the experiences of McBee [31], Hsiung et al. [32] and Ernst et al. [30], who have developed and/or tested mindfulness-related interventions for this target group.

These principles together establish the foundational basis for the initial exercise framework, creating a structure upon which subsequent participatory enhancements were thoughtfully implemented.

## Results

### Step 1: theory- and evidence-based intervention draft

Based on the activities described in Step 1, we established a repertoire of 26 exercises in eight modules with additional introduction and ending as presented in Table 1.

### Step 2: testing with target group representatives

The logs of mini focus groups pertaining to the efficacy of the 26 exercises distributed in eight modules are provided in German language in Appendix 1b, and summarized in the following. The feedback exhibited considerable variance. Within the initial module addressing stress and stress management, discourse emerged regarding the exercise ‘goal establishment’, as participants perceived the goals set within the institutional framework as constraining. Conversely, ‘energy sources’ was received positively, with individual sources such as sleep, interpersonal discourse, and rituals being cited. The exercise entitled ‘New and Good’ provoked cognitive overload, prompting suggestions for its execution at a decelerated pace and with frequent intermissions. Feedback from the subsequent module, focusing on relaxation techniques, revealed ‘diaphragmatic breathing’ as a well-received exercise, employed for stress reduction purposes. Additionally, the participants expected a high efficacy of the ‘fist exercise’ in interrupting ruminative ideation patterns. Meditation practices and the elicitation of the ‘relaxation response’ were acknowledged as instrumental tools in fostering states of relaxation. Moving to the third module emphasizing physical activity, the ‘body scan’ exercise emerged as a favorably perceived intervention, although its execution without guidance posed challenges to participants. Moreover, physical Yoga and Qi Gong were perceived as pleasant and helped the participants to start the day more actively. The walking meditation, on the other hand, was perceived as difficult by some participants, especially for those with motor impairments. The SARW (stop, breathe, reflect, choose) exercise was understood verbally, but was not understood internally. In module four, focusing on nutritional aspects, the raisin exercise elicited mixed responses, with both favorable and adverse reactions noted. Attention to hydration patterns emerged as pivotal, albeit with reported challenges among certain participants in meeting recommended fluid intake levels. The exercise requiring the identification of ‘20 things—> 3 things’ proved challenging, with hesitancy noted in sharing intimate facets within the group. The subsequent module, centered on social networks and relational dynamics, received commendation for prompting introspection regarding personal connections. Acknowledgment of familial bonds and individual pursuits in later life stages was notably underscored. In module six (language and expression), the postcard exercise encountered challenges among some participants, particularly those grappling with motor impairments or negative ideation patterns. Similarly, the Loving-Kindness Meditation elicited adverse emotional responses in some participants, concurrently fostering reflective introspection. Module seven, elucidating self-help strategies and mechanisms of self-healing, facilitated contemplation on existential themes and personal values. The significance of humor and laughter was emphasized, notwithstanding the impediment posed by facial masks (during the pandemic) on non-verbal communication. Concluding in the eighth module focusing on relapse prevention, participants delineated personal goals and coping strategies, with notable instances of difficulty encountered in answering specific questions as part of the exercise.

### Steps 3 and 4: online pre-test and survey with stakeholders

#### Participants of online pre-test

In the pre-test for the survey, 15 complete datasets were collected. The dataset consists of answers by one nursing facility manager, one nursing professional, one member of the project advisory board, seven scientists in the field of health promotion/MBM and five other persons, including one physiotherapist/research assistant in MBM, one psychology student and three student assistants of the IGVF.

#### Participants of online survey

In total, we collected 12 valid records from the online stakeholder survey. Among these participants were one nursing professional, three persons from the social service of a nursing facility, three members of the project advisory board, one scientist in the field of health promotion/MBM and four other persons, whose role or profession could not be identified.

#### Quantitative results of online pre-test and online survey

The quantitative findings from the online pre-test and subsequent online survey are detailed in Appendix 3. The results from the first module on stress and stress management indicated that participants believed the exercises were largely suitable for the target group without significant adaptations. For instance, the exercise ‘New and Good’ was deemed suitable by 75% of participants. However, the ‘Setting Goals’ exercise required specific adjustments, as only 33% of participants felt the target group could follow it without additional guidance. These observations were confirmed in the online survey, where 57% of participants indicated that further adjustments were needed for the ‘Setting Goals’ exercise. In the second module on relaxation, the pre-test results suggested minimal adjustments were necessary, with most exercises being considered appropriate for the target group. Nevertheless, specific exercises like ‘Fist’ (40%) and ‘Minis’ (36%) required some modifications. The online survey corroborated these findings, indicating the need for slight adjustments to better align these exercises with the target group’s capabilities. For the third module on exercise, the pre-test showed a high level of comprehensibility, with exercises like the Qi Gong breathing exercise rated suitable by 89% of participants. However, the yoga exercises required significant adaptations, as only 22% of participants found them appropriate without changes. The online survey supported these results, with a notable 75% of participants deeming the ‘Walking Meditation’ unsuitable and 50% indicating that the ‘Body Scan’ needed adjustments. In the nutrition module, pre-test participants generally felt that the exercises were suitable for the target group, except for the ‘Mediterranean Diet’ exercise, which required modifications. The online survey echoed these findings, with most exercises being suitable, though the ‘20 Things—> 3 Things’ exercise was highlighted for needing improvements. The social networking module's pre-test results indicated that the exercises were appropriate and required no adjustments. However, the online survey revealed that 67% of participants thought modifications were necessary to better tailor the exercises to the target group. For the sixth module on language and expression, pre-test feedback suggested that the exercises were suitable. Nonetheless, the online survey indicated the need for adjustments, particularly in the ‘Loving-Kindness Meditation’ and ‘Postcard’ exercises, to better fit the target group. Finally, the module on self-help and self-healing were well received in both the pre-test and online survey, with participants agreeing that the exercises were effectively designed and delivered for the target group.

#### Qualitative results

The deductive-inductive approach to analyzing the open-field questions in the pre-test and online survey revealed valuable insights into specific problems identified by the participants. The category system including sub-category definitions and anchor examples are summarized in Appendix 4.

Regarding the first module, the importance of addressing cognitive, emotional, and physical concerns during goal setting has been emphasized. Clear explanations and examples are essential, as is the use of easily understandable language. Appropriate presentation and technical support are crucial for effective implementation. The ‘Energy Sources’ exercise should consider individuals living alone and include a clear definition of energy sources. For the ‘New and Good’ exercise, cognitive challenges faced by individuals with depression must be taken into account. Feedback for the relaxation module indicates a need for clear instructions and adaptations for different groups, suggesting that simpler wording and clear labeling are necessary.

The feedback for the relaxation module highlights several concerns regarding the ‘Fist’ exercise, diaphragmatic breathing, the meditation relaxation response, and the ‘Minis’. For the ‘Fist’ exercise, participants raised issues related to the video length, physical effort, and coordination, particularly for individuals who are paralyzed or bedridden. Specific task instructions, simpler language, and clear guidance are recommended to ensure successful execution. The suggestion to perform the task mentally was included. Regarding diaphragmatic breathing, cognitive challenges and physical difficulties were noted, with a recommendation to adapt the exercise for lying down. The meditation relaxation response may be too lengthy and cognitively demanding for some participants; therefore, simplified examples and easier wording are suggested. For the ‘Minis’, there are concerns about participants’ ability to remember previous exercises and their names, prompting recommendations for summaries or keywords. Additionally, it is advised to divide the exercises into separate videos and provide transparent information about the length and implementation process.

The feedback for the movement module addresses various exercises, including meditation, yoga, Qi Gong, walking meditation, and the exercise SARW. Participants expressed cognitive, emotional, and physical concerns, highlighting challenges related to memory, concentration and physical fitness. Recommendations include clear instructions, shortened exercises, alternative positions, and supportive guidance. Technical improvements such as better audio quality and engaging presentation are also suggested. Additionally, the use of inclusive language and simpler wording are emphasized as important aspects of the feedback.

The feedback for the nutrition module highlights concerns about participants’ ability to remember the exercises in everyday life and to apply them with specific health challenges, such as swallowing difficulties. Recommendations include suggesting alternative foods and providing clear instructions for the exercises, as well as using simpler language and implementing technical improvements. Similar concerns were raised for the mindful eating exercise, particularly regarding the risk of choking for individuals with certain conditions and the need for personalized dietary considerations. It is advised to include alternative food options. For the ‘Treasure Chest’ exercise, participants expressed worries about its abstract nature and potential difficulties for individuals with cognitive impairments. Recommendations include explaining the exercise’s value and providing concrete examples, as well as placing it at the end of a thematic block. Concerns about concentration and emotional issues were noted for the ‘Imagination Kitchen’. Suggestions include using simpler wording and offering alternative exercise environments to address these potential difficulties.

In the social network module, participants provided feedback on the ‘Social Atom’ exercise. They expressed difficulties with the abstract nature of the exercise and the drawing component. Additionally, emotional concerns were raised for individuals feeling lonely, along with physical challenges during implementation. Recommendations include providing a clearer explanation of the exercise’s value, offering additional examples, and emphasizing that different outcomes are acceptable. Technical improvements and support for the drawing process were also suggested.

In the language and expression module, feedback to two exercises was evaluated: the ‘Postcard’ and ‘Loving-Kindness Meditation’. For the postcard exercise, participants expressed concerns about the unfamiliarity of the task, emotional barriers, and physical limitations. Recommendations include providing a clearer explanation of the exercise’s value, offering additional examples, and suggesting alternative implementation options. Regarding the ‘Loving-Kindness Meditation’, participants noted cognitive challenges, emotional concerns, and difficulties for individuals with cognitive impairments. It is recommended to simplify the exercise, address negative emotions, and shorten its duration to enhance accessibility.

In the self-help and self-healing module, feedback regarding two exercises are evaluated: ‘Meaning and Purpose’ and ‘Humor’. For the ‘Meaning and Purpose’ exercise, participants raised concerns about the intellectual demands and emotional burdens, particularly among depressed seniors. Recommendations include providing additional examples and using simpler language. The ‘Humor’ exercise did not present specific concerns; however, participants found the gestures used during the presentation to be challenging.

In the relapse prevention module, participants expected cognitive concerns about feeling overwhelmed by extensive exercises and difficulties with memory recall. It is recommended to provide a brief summary of each exercise. For the ‘Self-Regulation Recipes’ exercise, participants struggled to remember it based on its name, leading to the suggestion for an accompanying video with short descriptions of the exercises. Additionally, it was noted that older individuals in nursing homes are often advanced in age and have various impairments, which can affect the acceptance and execution of the exercises. General recommendations include avoiding technical jargon, standardizing labels, and using engaging language that respects the target population.

### Steps 5 and 6: discussion of the results by the project team and feedback from advisory board & funding organization

In summary, a large amount of feedback coincides with the basic assumptions of the feasibility of mindfulness-based and -informed interventions with seniors. Concerns were taken seriously, especially in the case of exercises that received multiple feedback that they might be too cognitively, emotionally or physically overloading for the target group. 

#### Global adjustments

Global adjustments are presented in Table 2 and were implemented for each exercise where contextually appropriate. The global adjustments span different aspects, including content changes, video presentation, sound quality, technical support, wording, formatting, documentation, surroundings, inclusion and exclusion criteria, and memory aids.

**Table 2:** Handling of General Feedback (Transfer to Research Design, Multiple/All Exercises)

Content	Changes	Consider by	Comment
**Gong**	Use of a different Gong, a deeper gong only after the introductory words	Video production	
	Clear distinction between introduction and practice	Video production	
	Only play once	Video production	Gong one time at the beginning and one time at the end
	Unify across all exercises	Video production	
**Independence/possibility of support**	Possible assistance or other options highlighted	Instruction/Manual	
**Video/image material for assistance only**	Note that the image is only intended as an aid and does not need to be considered depending on your preference (close your eyes)—copy the text into the introduction to the app and the exercises	App	Since some exercises have visual instructions in the video, this cannot be implemented consistently. The possibility of “closing your eyes” is pointed out in the corresponding exercises/instructions
**Add greeting/introduce trainer**	Add greeting	Video production	Separate video with a short introduction of the speaker and the project, in coordination with funder
	Introduce trainer	Video production	See above
**Presentation in the video**	Tempo	Video production	
	Speak animatedly	Video production	
	Tone of voice and temperament	Video production	
	Expand facial expressions	Video production	
	Smile	Video production	
	Camera eye contact	Video production	
	Expand gestures	Video production	
	Breaks	Video production	
	image detail (person not too far away)	Video production	
**Sound quality**	Higher sound quality	Video production	
**Technical support**	Technical introduction required (speaking into the app independently is difficult)	Instruction/Manual	Also supported by accompanying people
**Make regular offers to practice**	Allow repetition/practice/space for change (e.g., give as homework)	App, Accompaniment	It is suggested several times and is also technically supported
	Motivate to practice	Instruction/Manual	
**Font**	Larger font size: prefer black writing (decreasing ability to recognize colors)	App, Intervention manual	Colors are determined by vdek CI. Font size can technically be individually adjusted. → relevant for manual
**Recognize and maintain your own boundaries as a principle**	This means that exercises can be stopped or paused at any time	Wording/Text	
**Position of the exercise**	Mention in the instructions if the exercise can be done differently than shown (sitting/lying down)	Instruction/Manual	
**Consider hearing difficulties**	Explain volume regulation	App, Instruction/Manual	Hearing can technically be individually adjusted. → relevant for manual
	In group intervention: if necessary, go closer to individual people again	Group, Instruction/Manual	
**Documentation in group intervention**	Trainers should note if someone performed the exercise significant differently (deviations)	Group, Instruction/Manual	
	Special features: e.g., when someone is sleeping	Group, Instruction/Manual	
**Surrounding**	Quiet surrounding necessary	Group, Instruction/Manual	
**Foreign words/technical terms**	Avoid foreign words/technical terms (App, Podcast etc.)	Texts	“App” will continue to be used because this is assumed to be known to participants with an app
**Inclusion/exclusion criteria**	Exclude people with depression; Geriatric Depression Scale (GDS)	Study	Include further questions from the geriatric depression scale in the preliminary interview
	Clarify Suicidality	Study	Preliminary interview before study
**Reduction**	Shortening of the exercises – > equalization, sometimes offered in two lengths	Video production	
	Group sessions (relevant for group instruction): Net exercise time: 45/50 min + organization time (choose a place,…) – > 75 min (Tobias Esch's assessment, based on personal exchange with John Kabat-Zinn)	Group, Instruction/Manual	
**Memory**	Provide memory cards for repeating the exercise (link actions to everyday life + memories)	Starter package for study	

Content changes involve modifications to the use of the Gong, the introduction and practice phases, and the standardization of exercises across sessions. Additionally, options for assistance and alternatives are highlighted, and video/image material is provided solely as aids for visual learners. Greetings are incorporated into sessions, and trainers are introduced to establish rapport. In terms of video presentation, emphasis is placed on the tempo, tone, and demeanor of the presenter, with instructions to speak animatedly, maintain eye contact, and employ facial expressions and gestures effectively. Breaks and optimal camera positioning are also addressed. Attention is given to improving sound quality and providing technical support for participants who may require assistance with navigating the app independently. Wording and formatting recommendations include larger font sizes and black writing for readability, along with guidance for participants to recognize and respect their own boundaries during exercises. Instructions also clarify alternative positions for exercises and address volume regulation for participants with hearing difficulties. Procedures for documenting deviations during group interventions and ensuring a quiet and conducive environment for practice are outlined.

Protocols are established to exclude individuals with depression, assessed through the Geriatric Depression Scale (GDS) [64], German version: [65] and to address suicidality sensitively if it arises during sessions. Strategies for shortening exercises and providing memory aids for repetition are suggested, with the goal of facilitating engagement and retention of the intervention content. Overall, these adjustments are designed to optimize the intervention experience for participants, ensuring that it is tailored to their needs and conducive to positive outcomes.

#### Adjustments to specific exercises

In addition to the global adjustments, specific adaptations also stem from the feedback. In Appendix 5, we provide the detailed list of adjustment to each of the initial exercises. This list is summarized in the following.

In the first module, additional examples and questions were used when setting goals and identifying sources of strength. The ‘New and Good’ exercise is designed to encourage regular practice. In Module 2, adjustments were made to the relaxation exercises, including clearer guidance on diaphragmatic breathing and the ‘Fist’ exercises as well as a more flexible meditation duration. Establishing a daily routine is now recommended after certain exercises, and ‘Minis’ for self-practice have been added. Module 3 on physical activity has been improved with more detailed instructions for the ‘Body Scan’, Yoga and Qi Gong exercises. In addition, a note has been added to the ‘Walking Meditation’, indicating that walking aids can also be used. In Module 4 on nutrition, mindfulness exercises such as the raisin exercise and water intake have been updated to include specific restrictions or allergies. In Module 5 (Social Network, Friends), an additional example and pauses have been added to the ‘Social Atom’ exercise. Examples have been added to the first exercise in Module 6, Language and Expression, and the opportunity to draw a picture has been added. In ‘Loving-Kindness-Meditation’, minor adjustments have been made to the text. In the exercise ‘Meaning and Purpose’ from Module 7, pauses have been added and the wording has been simplified. In the last module (Relapse Prevention), the name of the exercise was standardized in the exercise ‘Self-Regulation Recipes’ and adjustments were made to the wording in the exercise. A conclusion and a link to the exercise overview was provided.

### Step 7: app development

A user-centered design was the goal of the project. Monthly meetings between the project team and the app-developer took place in the final stage of the project. Initially, two-design approaches were presented by the app developers and discussed among research team members and during a subsequent meeting with the three representatives of the target group. Afterwards, a first app-version was presented to the advisory board in order to gain further insights, suggestions, and critical evaluations. After reiteration and further discussion in the project team, it was finalized for a feasibility study [58].

## Discussion

### Summary of results

The aim of this study was to use PBCD methods to facilitate the translation of meaningful stakeholder experiences into the design of an on-site and digitally delivered MII for health promotion in nursing homes, called ‘silBERN’. The PBCD process for the intervention involved multiple stakeholders, including the target group, their relatives, nurses and other experts from nursing homes, health insurance representatives and app developers. The iterative process comprised several key steps: theory-based selection of mind–body exercises by the project team, informal discussions with intervention experts, semi-structured interviews with the target audience (*n* = 3) during 12 sessions, pre-test of the exercises through an online survey with researchers (*n* = 15), online survey with project partners, nursing facility experts, caregivers, and the general public (*n* = 12). Qualitative feedback was assessed through deductive-inductive content-structuring analysis. For the digital version, the app developers created two front-end application examples, of which one was further refined based on regular feedback from the target audience and the research team.

The intervention draft includes 26 exercises across eight modules focusing on well-being, with feedback from mini focus groups with the target population indicating significant variability in responses. Participants found goal-setting constraining but positively identified personal energy sources. Exercises like diaphragmatic breathing and body scans were well-received, while mental exercises tended to cause cognitive overload. Challenges arose with certain activities, particularly for those with motor impairments, and emotional responses varied widely in language and expression exercises. In the online pre-test and online survey, a total of 27 datasets were collected from various professionals, including nursing staff and researchers, revealing that most exercises in the first module on stress management were suitable. Subsequent modules showed similar trends, with exercises like Qi Gong rated highly suitable, while yoga and walking meditation required significant modifications. Overall, the surveys indicated a consensus that while many exercises were appropriate, targeted adjustments were necessary to enhance accessibility and fit for the participant group. The qualitative analysis of open-field responses from the pre-test and online surveys identified several key concerns and recommendations for the intervention modules. Global adjustments included improvements in content presentation, video quality, sound clarity, and readability, alongside ensuring supportive environments for participants. Emphasis was placed on establishing rapport, using appropriate language, and standardizing exercise protocols while incorporating memory aids and strategies for navigating the intervention effectively. Participants emphasized the need for clear explanations and accessible language in goal-setting, highlighting the importance of addressing cognitive, emotional, and physical challenges. Specific exercises, such as the ‘Fist’ and ‘Diaphragmatic Breathing’, required adaptations to accommodate physical limitations, while the ‘New and Good’ exercise needed adjustments to support individuals with depression. Additionally, there were calls for technical improvements, alternative food options in the nutrition module, and a clearer understanding of the exercises’ value to enhance participant engagement and efficacy. These comprehensive adjustments aim to optimize the overall experience and outcomes for participants. Several pieces of feedback from open-field questions were discussed but ultimately disregarded by the research team due to ambiguity or complexity. The final app version was designed with user-centered principles, incorporating insights from team discussions and stakeholder evaluations before being finalized for feasibility testing. Finally, based on the feedback, the inclusion restrictions for the RCT were amended with respect to mental health, i.e. participants with depression were planned to be excluded from participation.

### Advantages and limitations of the person-based co-design approach

The implemented steps of this person-based co-design approach comprise a participatory process that has used previous methods for intervention development in a flexible manner. It has been recommended by experts that it is useful to pursue a flexible approach in order to fit their individual problems and context [66]. The conglomeration of our methods applied has led to a significant change of initial content and delivery of exercises. In summary, all process elements proved useful. The mini focus groups conducted with the target population are highly relevant within the person-based co-design approach. They provided diverse feedback, indicating a range of needs and preferences, which is crucial for tailoring the intervention effectively. Participants identified specific constraints, such as the impact of institutional frameworks on goal establishment, and offered mixed responses to various exercises, highlighting both positive aspects and areas needing improvement. Insights on contextual relevance, such as the importance of hydration and personal connections, further ensured the interventions resonate with participants’ lived experiences. Additionally, the focus groups revealed necessary adaptations of exercises to accommodate varying abilities and uncovered emotional responses that inform safe intervention design. Overall, the focus groups fostered reflection on personal values and relationships, addressing multiple dimensions of well-being and aligning with the principles of person-centered care.

Participants in the pre-test survey, primarily consisting of researchers from various scientific disciplines, were less critical of the exercises’ suitability. In contrast, the online survey group was more diverse in terms of professional backgrounds and included individuals who were closer to the target group. This disparity in perspectives highlights the importance of involving a range of stakeholders in the intervention development process. Engaging professionals who regularly interact with the target group, in addition to including the target group itself, is essential for ensuring the relevance and effectiveness of the interventions.

The detailed feedback based on the open-field questions in the pre-test and online surveys is highly relevant for intervention development as it provides essential insights into the needs and challenges of the target population. It emphasizes the importance of tailored adaptations to address cognitive, emotional, and physical abilities, ensuring the intervention is user-friendly and engaging. Understanding emotional barriers promotes psychological safety, which is crucial for vulnerable groups. Additionally, the feedback suggests practical improvements in clarity, simplicity, and technical aspects, facilitating effective implementation in real-world settings. Addressing memory challenges directly enhances the feasibility of daily use, while the focus on inclusive language and personalized approaches ensures accessibility and fosters a sense of belonging.

Representatives from the project team, app developers, and health insurance representatives convened regularly to ensure constant collaboration. These meetings served to review progress, address challenges, and align the development trajectory with the project’s goals. This iterative approach enabled quick adjustments, leading to the timely incorporation of feedback and enhancements.

A critical assessment of our approach within the participation model by Wright et al. [67], defining the levels non-participation (instrumentalization, instruction), preliminary stages of participation (information, consultation, inclusion), participation (co-determination, partial decision-making power, decision-making authority), and beyond participation (self-organization), reveals that we primarily achieved a preliminary stage of participation through including and consulting different target groups. However, the advisory board provided partial co-determination. The process was eventually not democratic but was guided by the evaluation criteria of the research team. This nuanced positioning highlights the extent and limitations of our participatory efforts, indicating that while we incorporated elements of inclusion and partial co-determination, full democratic participation was not realized. However, considering the limited research landscape on including older adults in interventions development, our study aims to contribute initial insights into the process.

Despite this and the positive outcomes described above, several challenges were encountered during the PBCD approach, as previous studies on the development of health care interventions [57, 68–72] have highlighted. One significant issue was the ambiguity in feedback from the mini focus groups and surveys, which was often unclear or contradictory, making it difficult to determine specific action points. For example, there were conflicting opinions on including certain concepts like “receiving support” and terms such as “courage to live” and “strength,” leading to these suggestions being ignored. This ambiguity can hinder the refinement process and potentially overlook valuable insights. The complexity of the exercises also posed a challenge, as several were perceived as too cognitively, emotionally, or physically demanding for the target group. Addressing these concerns necessitated extensive modifications, indicating that the initial exercise designs may not have been adequately aligned with the capabilities of the target population. Technical and practical limitations further impacted the implementation of feedback. Emotional and psychological barriers emerged as significant issues, with exercises like the ‘Loving-Kindness Meditation’ and ‘Postcard’ eliciting adverse emotional responses from some participants, particularly those with cognitive impairments or negative ideation patterns. These reactions highlight the potential emotional risks associated with certain mindfulness practices, necessitating careful consideration and adaptation to ensure psychological safety. Furthermore, logistical and implementation challenges included the need for detailed instructions, alternative positions, and technical support, which highlights the practical difficulties in implementing the intervention in real-world settings. Ensuring that participants can independently navigate the app and effectively engage with the exercises requires substantial support and clear guidance, which may not always be feasible.

The overall process was time-consuming, affecting both the research team and the involved stakeholders. This is particularly challenging in the healthcare context, where healthcare professionals typically face high workloads [69]. Moreover, participating patients often do not see immediate benefits from development projects, which can negatively impact their motivation and engagement [51].

Overall, while the PBCD approach provided valuable insights and led to significant improvements in the intervention, several challenges persist in ensuring the feasibility, accessibility, and emotional safety of mindfulness-based and -informed interventions for residents in nursing homes. Further experimental testing is necessary to determine the extent to which such approach enhances usability and patient outcomes in clinical practice [51]. Finally, it should be noted that the literature drawn upon to discuss the results is part of a nascent field of research, indicating a substantial need for further studies to address these and other emerging challenges. Finally, through the implementation of the feasibility study, additional development needs emerged from practice [73].

## Conclusion

This study demonstrates the effectiveness of the person-based co-design approach in developing tailored mindfulness-based and -informed interventions for seniors. By actively involving stakeholders, including healthcare professionals and the target population, the research identified key needs and preferences that significantly enhanced the interventions’ relevance and usability. Despite facing challenges such as high workloads for healthcare providers and the potential lack of immediate benefits for participating seniors, the insights gained led to meaningful improvements in the program and app design. Further experimental testing is necessary to assess the long-term impact of these interventions on usability and patient outcomes within clinical practice. Ultimately, this research underscores the importance of collaborative approaches in fostering accessible and emotionally safe interventions for vulnerable populations.

## Supplementary Information

Supplementary Material 1.

Supplementary Material 2.

Supplementary Material 3.

Supplementary Material 4.

Supplementary Material 5.

## Data Availability

Original data can be requested from the corresponding author.

## Abbreviations

MBM: Mind-Body Medicine
MBEC: Mindfulness-Based Elder Care
MBSR: Mindfulness-based stress reduction
PBCD: Person-based co-design
RCT: Randomized controlled trial
IGVF: Institute of Integrative Health Care and Health Promotion

## References

[1] Dow B, Lin X, Tinney J, Haralambous B, Ames D. Depression in older people living in residential homes. Int Psychogeriatr. 2011;23(5):681–99. 10.1017/S1041610211000494. 21429283 10.1017/S1041610211000494

[2] Ophey A, Brijoux T, Conrad A, Folkerts A-K, Zank S, Kalbe E. Cognition in people aged 80 years and older: determinants and predictors of change from a population-based representative study in Germany. J Frailty Aging. 2023;12(3):189–97. 10.14283/jfa.2023.20. 37493379 10.14283/jfa.2023.20

[3] Stoppe G. Depressionen im Alter. Bundesgesundheitsbl Gesundheitsforsch Gesundheitsschutz. 2008;51(4):406–10. 10.1007/s00103-008-0508-7. 10.1007/s00103-008-0508-7 18345472

[4] Wöhl C, Siebert H, Blättner B. Interventionen zur Förderung der körperlichen Aktivität in Pflegeheimen : Systematische Übersicht der Wirksamkeit universeller Prävention. Z Gerontol Geriatr. 2017;50(6):475–82. 10.1007/s00391-016-1158-2. 27966009 10.1007/s00391-016-1158-2

[5] GKV-Spitzenverband. Leitfaden Prävention in stationären Pflegeeinrichtungen nach § 5 SGB XI. 2018.

[6] Grossman P. Mindfulness: awareness informed by an embodied ethic. Mindfulness. 2015;6(1):17–22. 10.1007/s12671-014-0372-5.

[7] Kabat-Zinn J. Gesund durch Meditation: Das große Buch der Selbstheilung mit MBSR. München: Knaur; 2013.

[8] Esch T. Die neuronale Basis von Meditation und Achtsamkeit. SUCHT. 2014;60(1):21–8. 10.1024/0939-5911.a000288.

[9] Hölzel BK, Lazar SW, Gard T, Schuman-Olivier Z, Vago DR, Ott U. How does mindfulness meditation work? Proposing mechanisms of action from a conceptual and neural perspective. Perspect Psychol Sci. 2011;6(6):537–59. 10.1177/1745691611419671. 26168376 10.1177/1745691611419671

[10] Crane RS, Brewer J, Feldman C, Kabat-Zinn J, Santorelli S, Williams JMG, Kuyken W. What defines mindfulness-based programs? The warp and the weft. Psychol Med. 2017;47(6):990–9. 10.1017/S0033291716003317. 28031068 10.1017/S0033291716003317

[11] Brinkhaus B, Esch T, editors. Integrative Medizin und Gesundheit. Berlin: MWV Medizinisch Wissenschaftliche Verlagsgesellschaft; 2021.

[12] Michaelsen MM, Graser J, Onescheit M, Tuma MP, Werdecker L, Pieper D, Esch T. Mindfulness-based and mindfulness-informed interventions at the workplace: a systematic review and meta-regression analysis of RCTs. Mindfulness. 2023; 10.1007/s12671-023-02130-7. 10.1007/s12671-023-02130-7 PMC10172073 37362186

[13] Seligman MEP, Csikszentmihalyi M. Positive psychology: an introduction. Am Psychol. 2000;55(1):5–14. 10.1037/0003-066X.55.1.5. 11392865 10.1037//0003-066x.55.1.5

[14] Sutipan P, Intarakamhang U, Macaskill A. The impact of positive psychological interventions on well-being in healthy elderly people. J Happiness Stud. 2017;18:269–91. 10.1007/s10902-015-9711-z.

[15] Kabat-Zinn J. An outpatient program in behavioral medicine for chronic pain patients based on the practice of mindfulness meditation: theoretical considerations and preliminary results. Gen Hosp Psychiatry. 1982;4(1):33–47. 10.1016/0163-8343(82)90026-3. 7042457 10.1016/0163-8343(82)90026-3

[16] Alsubaie M, Abbott R, Dunn B, Dickens C, Keil TF, Henley W, Kuyken W. Mechanisms of action in mindfulness-based cognitive therapy (MBCT) and mindfulness-based stress reduction (MBSR) in people with physical and/or psychological conditions: a systematic review. Clin Psychol Rev. 2017;55:74–91. 10.1016/j.cpr.2017.04.008. 28501707 10.1016/j.cpr.2017.04.008

[17] Khoury B, Sharma M, Rush SE, Fournier C. Mindfulness-based stress reduction for healthy individuals: a meta-analysis. J Psychosom Res. 2015;78(6):519–28. 10.1016/j.jpsychores.2015.03.009. 25818837 10.1016/j.jpsychores.2015.03.009

[18] Kim SM, Park JM, Seo H-J, Kim J, Noh J-W, Kim HL. Effects of mindfulness-based stress reduction on adults with sleep disturbance: an updated systematic review and meta-analysis. BMJ Open. 2022;12(11): e058032. 10.1136/bmjopen-2021-058032. 36332952 10.1136/bmjopen-2021-058032 PMC9639069

[19] Lehrhaupt LM, Meibert P. Stress bewältigen mit Achtsamkeit: Zu innerer Ruhe kommen durch MBSR* - *Mindfulness-Based Stress Reduction. 6th ed. München: Kösel; 2014.

[20] Kabataş Yıldız M, Orak OS. The effect of the mindfulness-based stress reduction program on the level of perceived stress and geriatric depression in older adults: a randomised controlled study. Psychogeriatrics. 2023;23(2):261–72. 10.1111/psyg.12929. 36594217 10.1111/psyg.12929

[21] Parra DC, Wetherell JL, van Zandt A, Brownson RC, Abhishek J, Lenze EJ. A qualitative study of older adults’ perspectives on initiating exercise and mindfulness practice. BMC Geriatr. 2019;19(1): 354. 10.1186/s12877-019-1375-9. 31865906 10.1186/s12877-019-1375-9 PMC6927182

[22] Farb NAS, Murchison J, Madan R, Goldberg H, Grief C, Conn D, Khatri N. Mindfulness-based stress reduction interventions for mood in older adults: how do qualitative experiences inform clinical response? Mindfulness. 2021;12(7):1733–47. 10.1007/s12671-021-01636-2.

[23] Gentile C, Starnino L, Dupuis G, D’Antono B. Mindfulness-based stress reduction in older adults at risk for coronary artery disease: a pilot randomized trial. Clin Gerontol. 2022;45(2):272–86. 10.1080/07317115.2021.1887421. 33719899 10.1080/07317115.2021.1887421

[24] Lindsay EK, Creswell JD, Stern HJ, Greco CM, Walko TD, Dutcher JM, et al. Mindfulness-based stress reduction increases stimulated IL-6 production among lonely older adults: a randomized controlled trial. Brain Behav Immun. 2022;104:6–15. 10.1016/j.bbi.2022.05.001. 35550854 10.1016/j.bbi.2022.05.001 PMC9646928

[25] Lindsay EK, Marsland AL, Cole SW, Dutcher JM, Greco CM, Wright AGC, et al. Mindfulness-based stress reduction reduces proinflammatory gene regulation but not systemic inflammation among older adults: a randomized controlled trial. Psychosom Med. 2024;86(5):463–72. 10.1097/PSY.0000000000001264. 37982547 10.1097/PSY.0000000000001264 PMC11098967

[26] Lenze EJ, Voegtle M, Miller JP, Ances BM, Balota DA, Barch D, et al. Effects of mindfulness training and exercise on cognitive function in older adults: a randomized clinical trial. JAMA. 2022;328(22):2218–29. 10.1001/jama.2022.21680. 36511926 10.1001/jama.2022.21680 PMC9856438

[27] Berk L, van Boxtel M, van Os J. Can mindfulness-based interventions influence cognitive functioning in older adults? A review and considerations for future research. Aging Ment Health. 2017;21(11):1113–20. 10.1080/13607863.2016.1247423. 27827541 10.1080/13607863.2016.1247423

[28] Li SYH, Bressington D. The effects of mindfulness-based stress reduction on depression, anxiety, and stress in older adults: a systematic review and meta-analysis. Int J Mental Health Nurs. 2019;28(3):635–56. 10.1111/inm.12568. 10.1111/inm.12568 30656813

[29] Moss AS, Reibel DK, Greeson JM, Thapar A, Bubb R, Salmon J, Newberg AB. An adapted mindfulness-based stress reduction program for elders in a continuing care retirement community: quantitative and qualitative results from a pilot randomized controlled trial. J Appl Gerontol. 2015;34(4):518–38. 10.1177/0733464814559411. 25492049 10.1177/0733464814559411 PMC5973835

[30] Ernst S, Welke J, Heintze C, Gabriel R, Zöllner A, Kiehne S, et al. Effects of mindfulness-based stress reduction on quality of life in nursing home residents: a feasibility study. Forsch Komplementmed. 2008;15(2):74–81. 10.1159/000121479. 18496020 10.1159/000121479

[31] McBee L. Mindfulness-based elder care: A CAM model for frail elders and their caregivers. New York, NY 10036: Springer Publishing Company, LLC; 2008.

[32] Hsiung Y, Chen Y-H, Lin L-C, Wang Y-H. Effects of mindfulness-based elder care (MBEC) on symptoms of depression and anxiety and spiritual well-being of institutionalized seniors with disabilities: a randomized controlled trial. BMC Geriatr. 2023;23(1):497. 10.1186/s12877-023-04220-6. 37596549 10.1186/s12877-023-04220-6 PMC10439662

[33] Hazlett-Stevens H, Singer J, Chong A. Mindfulness-based stress reduction and mindfulness-based cognitive therapy with older adults: a qualitative review of randomized controlled outcome research. Clin Gerontol. 2019;42(4):347–58. 10.1080/07317115.2018.1518282. 30204557 10.1080/07317115.2018.1518282

[34] Esch T, Esch S. Stressbewältigung. Mind-Body-Medizin, Achtsamkeit, Resilienz. 4th ed. Berlin: Medizinisch Wissenschaftliche Verlagsgesellschaft; 2024.

[35] Esch T, Stefano GB. The BERN framework of mind-body medicine: integrating self-care, health promotion, resilience, and applied neuroscience. Front Integr Neurosci. 2022;16: 913573. 10.3389/fnint.2022.913573. 35910341 10.3389/fnint.2022.913573 PMC9330052

[36] Finegood DT, Johnston LM, Steinberg M, Matteson CL, Deck P. Complexity, systems thinking, and health behavior change. Health Behavior Change in Populations. 2014:435–58.

[37] Leask CF, Sandlund M, Skelton DA, Altenburg TM, Cardon G, Chinapaw MJM, et al. Framework, principles and recommendations for utilising participatory methodologies in the co-creation and evaluation of public health interventions. Res Involv Engagem. 2019;5:2. 10.1186/s40900-018-0136-9. 30652027 10.1186/s40900-018-0136-9 PMC6327557

[38] Bishop FL, Fenge-Davies AL, Kirby S, Geraghty AWA. Context effects and behaviour change techniques in randomised trials: a systematic review using the example of trials to increase adherence to physical activity in musculoskeletal pain. Psychol Health. 2015;30(1):104–21. 10.1080/08870446.2014.953529. 25109300 10.1080/08870446.2014.953529

[39] Damschroder LJ, Aron DC, Keith RE, Kirsh SR, Alexander JA, Lowery JC. Fostering implementation of health services research findings into practice: a consolidated framework for advancing implementation science. Implement Sci. 2009;4:50. 10.1186/1748-5908-4-50. 19664226 10.1186/1748-5908-4-50 PMC2736161

[40] Murray E, Treweek S, Pope C, MacFarlane A, Ballini L, Dowrick C, et al. Normalisation process theory: a framework for developing, evaluating and implementing complex interventions. BMC Med. 2010;8:63. 10.1186/1741-7015-8-63. 20961442 10.1186/1741-7015-8-63 PMC2978112

[41] Robert G, Macdonald AS. Co-design, organizational creativity and quality improvement in the healthcare sector: ‘Designerly’ or ‘design-like’? In: Sangiorgi D, Prendiville A, eds. Designing for Service. Bloomsbury Publishing Plc; 2017. p. 117–130. 10.5040/9781474250160.ch-009.

[42] Tarquinio C, Kivits J, Minary L, Coste J, Alla F. Evaluating complex interventions: perspectives and issues for health behaviour change interventions. Psychol Health. 2015;30(1):35–51. 10.1080/08870446.2014.953530. 25140439 10.1080/08870446.2014.953530

[43] Bowen DJ, Kreuter M, Spring B, Cofta-Woerpel L, Linnan L, Weiner D, et al. How we design feasibility studies. Am J Prev Med. 2009;36(5):452–7. 10.1016/j.amepre.2009.02.002. 19362699 10.1016/j.amepre.2009.02.002 PMC2859314

[44] Vries D de, Kinsman J, Cremers L, Rios M, Takács J, Ciotti M, Tsolova S. Community engagement for public health events caused by communicable disease threats in the EU/EEA. Stockholm: ECDC; 2020.

[45] Rustage K, Crawshaw A, Majeed-Hajaj S, Deal A, Nellums L, Ciftci Y, et al. Participatory approaches in the development of health interventions for migrants: a systematic review. BMJ Open. 2021;11(10): e053678. 10.1136/bmjopen-2021-053678. 34697122 10.1136/bmjopen-2021-053678 PMC8548676

[46] Bergold J, Thomas S. Partizipative Forschungsmethoden: Ein methodischer Ansatz in Bewegung. 2012. 10.17169/fqs-13.1.1801.

[47] Robert G, Cornwell J, Locock L, Purushotham A, Sturmey G, Gager M. Patients and staff as codesigners of healthcare services. BMJ. 2015;350: g7714. 10.1136/bmj.g7714. 25670179 10.1136/bmj.g7714

[48] Springett J, Atkey K, Kongats K, Zulla R, Wilkins E. Conceptualizing Quality in Participatory Health Research: A Phenomenographic. Inquiry. 2016. 10.17169/fqs-17.2.2568.

[49] Israel BA, Coombe CM, Cheezum RR, Schulz AJ, McGranaghan RJ, Lichtenstein R, et al. Community-based participatory research: a capacity-building approach for policy advocacy aimed at eliminating health disparities. Am J Public Health. 2010;100(11):2094–102. 10.2105/AJPH.2009.170506. 20864728 10.2105/AJPH.2009.170506 PMC2951933

[50] Koshy E. Action research in healthcare. Los Angeles, Calif.: SAGE; 2011.

[51] Elbers S, van Gessel C, Renes RJ, van der Lugt R, Wittink H, Hermsen S. Innovation in pain rehabilitation using co-design methods during the development of a relapse prevention intervention: case study. J Med Internet Res. 2021;23(1): e18462. 10.2196/18462. 33470937 10.2196/18462 PMC7857944

[52] Yardley L, Morrison L, Bradbury K, Muller I. The person-based approach to intervention development: application to digital health-related behavior change interventions. J Med Internet Res. 2015;17(1):e30. 10.2196/jmir.4055. 25639757 10.2196/jmir.4055 PMC4327440

[53] Kok G, Schaalma H, Ruiter RAC, van Empelen P, Brug J. Intervention mapping: protocol for applying health psychology theory to prevention programmes. J Health Psychol. 2004;9(1):85–98. 10.1177/1359105304038379. 14683571 10.1177/1359105304038379

[54] McCurdie T, Taneva S, Casselman M, Yeung M, McDaniel C, Ho W, Cafazzo J. Mhealth consumer apps: the case for user-centered design. Biomed Instrum Technol. 2012. 10.2345/0899-8205-46.s2.49. 23039777 10.2345/0899-8205-46.s2.49

[55] Yardley L, Ainsworth B, Arden-Close E, Muller I. The person-based approach to enhancing the acceptability and feasibility of interventions. Pilot Feasibility Stud. 2015;1(1):37. 10.1186/s40814-015-0033-z. 27965815 10.1186/s40814-015-0033-z PMC5153673

[56] Giaccardi E, Stappers PJ. Research through Design. In: ; 2017. https://www.interaction-design.org/literature/book/the.

[57] Donetto S, Pierri P, Tsianakas V, Robert G. Experience-based co-design and healthcare improvement: realizing participatory design in the public sector. Des J. 2015;18(2):227–48. 10.2752/175630615X14212498964312.

[58] Uhl J, Schönfeld S, Meyer L, Reus A, Neumann C, Langer L, et al. (Digital) mind-body intervention to promote health and subjective well-being of residents in nursing homes: a cluster-randomized controlled pilot study. Gesundheitswesen. 2025. 10.1055/a-2517-8263. 39814069 10.1055/a-2517-8263

[59] Jilani H, Rathjen KI, Schilling I, et al. Handreichung zur Patient*innenbeteiligung an klinischer Forschung, Version 1.0. Universität Bremen; 2020. 10.26092/elib/1925.

[60] Bartholomew Eldredge LK, Markham CM, Kok G, Parcel GS, Fernandez ME, Ruiter RAC. Planning health promotion programs: An intervention mapping approach. San Francisco, CA: Jossey-Bass; 2016.

[61] Brüsemeister T. Qualitative Forschung: Ein Überblick. VS Verlag für Sozialwissenschaften; 2008.

[62] Kuckartz U. Mixed Methods: Methodologie. Forschungsdesigns und Analyseverfahren: Springer-Verlag; 2014.

[63] Antonovsky A, Franke A, Schulte N. Salutogenese: Zur Entmystifizierung der Gesundheit. dgvt Verlag; 1997.

[64] Yesavage JA, Brink TL, Rose TL, Lum O, Huang V, Adey M, Leirer VO. Development and validation of a geriatric depression screening scale: a preliminary report. J Psychiatr Res. 1983;17(1):37–49. 10.1016/0022-3956(82)90033-4. 10.1016/0022-3956(82)90033-4 7183759

[65] Gauggel S, Birkner B. Validität und Reliabilität einer deutschen Version der Geriatrischen Depressionsskala (GDS). Z Klin Psychol Psychother. 1999;28(1):18–27.

[66] O’Cathain A, Croot L, Duncan E, Rousseau N, Sworn K, Turner KM, et al. Guidance on how to develop complex interventions to improve health and healthcare. BMJ Open. 2019;9(8): e029954. 10.1136/bmjopen-2019-029954. 31420394 10.1136/bmjopen-2019-029954 PMC6701588

[67] Wright M, Block M, von Unger H. Partizipation der Zielgruppe in der Gesundheitsförderung und Prävention. In: Wright M, editor. Partizipative Qualitätsentwicklung in der Prävention und Gesundheitsförderung. Bern: Huber; 2010. p. 35–42.

[68] Boyd H, McKernon S, Mullin B, Old A. Improving healthcare through the use of co-design. N Z Med J. 2012;125(1357):76–87. 22854362

[69] Iedema R, Merrick E, Piper D, Britton K, Gray J, Verma R, Manning N. Codesigning as a discursive practice in emergency health services: the architecture of deliberation. J Appl Behav Sci. 2010;46(1):73–91. 10.1177/0021886309357544.

[70] Jamin G, Luyten T, Delsing R, Braun S. The process of co-creating the interface for VENSTER, an interactive artwork for nursing home residents with dementia. Disabil Rehabil Assist Technol. 2018;13(8):809–18. 10.1080/17483107.2017.1385102. 29037109 10.1080/17483107.2017.1385102

[71] Revenäs Å, Martin C, H Opava C, Brusewitz M, Keller C, Åsenlöf P. A Mobile Internet Service for Self-Management of Physical Activity in People With Rheumatoid Arthritis: Challenges in Advancing the Co-Design Process During the Requirements Specification Phase. JMIR Res Protoc. 2015;4(3):e111. 10.2196/resprot.4824. 10.2196/resprot.4824 PMC4704958 26381221

[72] Verbiest MEA, Corrigan C, Dalhousie S, Firestone R, Funaki T, Goodwin D, et al. Using codesign to develop a culturally tailored, behavior change mHealth intervention for indigenous and other priority communities: a case study in New Zealand. Transl Behav Med. 2019;9(4):720–36. 10.1093/tbm/iby093. 30388262 10.1093/tbm/iby093

[73] Meyer L, Kobs J, Reus A, Langer L. (Digital) mind-body intervention for residents in care facilities: mixed format of on-site and app intervention to combine benefits. THE MIND Bulletin on Mind-Body Medicine Research. 2024;5:6–7. 10.61936/themind/202410093.

