# Supplementary material for: Development of a (digital) mindfulness-informed intervention for older adults in nursing homes: description and reflection of a person-based co-design approach
Source: BMC Geriatr. 2025 Sep 25;25:703. doi: 10.1186/s12877-025-06223-x (PMC12462116; doi:10.1186/s12877-025-06223-x)
Supplement: Supplementary file 1 — Supplementary Material 1. [file 12877_2025_6223_MOESM1_ESM.docx]

**Appendix 1a: silBERN Semi-Structured Interview Guide for Mini Focus Groups**

**First Session:**

- Welcome and introduction
- Explanation of the study’s purpose and process
- Presentation of a video with exercise instructions (e.g., yoga, breathing exercises, walking meditation)
- Interview Questions:

1. Were you able to follow the trainer’s instructions?
2. How did you experience the execution of the exercise?
3. Would you like to add anything else?

**Second to Eleventh Sessions:**

- Welcome
- Presentation of a video with exercise instructions (e.g., yoga, breathing exercises, walking meditation)
- Interview Questions:

1. Were you able to follow the trainer’s instructions?
2. How did you experience the execution of the exercise?
3. Would you like to add anything else?

**Final Interview:**

In this session, we would like to reflect on the exercises one last time.

- Participants name their favorite exercises.
- Participants name the exercises they did not particularly enjoy (Do they remember them?).
- Brief follow-up on the remaining exercises: And how did you find exercise XYZ?

**Appendix 1b: Summary Logs of Mini Focus Groups in German**

**Modul 1, Stress und Stressbewältigung**

**Ziele setzen**

#### Feedback Praxispartner

Im Nachgang zur Übung diskutierten die Teilnehmenden eher das Thema Ziele an sich, als es jeweils für sich selbst im Sinne einer Übung umzusetzen. Es gab Bemerkungen, dass Ziele innerhalb der Einrichtung eingeschränkt sind.

Die Aufgabe wurde nicht in dem Sinne wie oben beschrieben angenommen; es blieb unklar, ob sie verstanden wurde. Jedoch rückte das Thema Ziele in den Mittelpunkt und wurde diskutiert.

**Kraftquelle**

#### Feedback Praxispartner

Besinnung auf die Kraftquellen wurde angenommen. Wie beabsichtigt wurde deutlich, dass jede/r seine/ihre eigenen Kraftquellen hat (genannt wurden: Schlafen; Gespräch mit jemandem, der Rat geben kann; Gespräch/Austausch mit Freunden, auch am Telefon; unterschiedliche Kraftquellen im Lebensverlauf, beispielsweise Gartenarbeit, Ehepartner:in; Rituale sind wichtig).

**Neues und Gutes**

#### Feedback Praxispartner

Die Übung schien kognitiv zu überfordern. Allerdings war die Übung in der Feedbacksitzung akustisch schwer zu verstehen; es solle langsamer und mit mehr Pausen gesprochen werden. Die Übung sollte mit weiteren Feedbackgebern aus der Zielgruppe durchgeführt werden.

**Modul 2, Entspannung**

### **Zwerchfellatmung**

#### Feedback Praxispartner

Die Übung wurde als sehr angenehm empfunden, sehr entspannend, Gedanken können umherwandern, abschalten war möglich; die Übung war bekannt aus der Reha und wird von einer Person der Zielgruppe bereits zum Einschlafen genutzt;
Die Übung wurde von einer Person kategorisiert als „wenn man Probleme mit der Atmung hat, ist diese Übung sinnvoll…“, es wurde auf die Erklärung, dass die Atmung mit unserem ganzen Sein in Verbindung steht und die Aufmerksamkeit auf einer gelenkten Atmung Stress mindern kann, nur kurz reagiert, dann wieder zurück zu der Idee, dass die Übung etwas für Menschen mit Atembeschwerden sei. Feedbackgebende haben den Eindruck, die Übung sei „nur unter Anleitung zu verinnerlichen“, „man macht es viel zu wenig“.

*Hinweis: Bei starker emotionaler Bewegung nach einer anderen Übung war die Zwerchfellatmung ein gutes Werkzeug, um wieder „Ruhe ins System zu bringen.“ Sie wird daher relativ früh im Kursplan bekannt gemacht*.

**Faustübung**

#### Feedback Praxispartner

Bewohner:innen konnten sehr gut folgen. Sie konnten aus ihrer Gedankenspirale aussteigen, Atmung hat sich beruhigt. Im Nachgespräch wurde etwas von Wut und Boxsack erzählt (was man „sonst nicht darf“). Durch die Entspannungsphase der Übung konnte der TN sich gut davon lösen. Anspannung beim Ballen der Faust wurde wahrgenommen.

Anspannung auch in anderen Körperteilen wurde wahrgenommen aber nicht im Nachhinein angesprochen.

Stimmlage im Video wurde positiv erlebt (schon wiederholt).

Langsam eingesprochen – verändert die Zeiträume von Anspannung und Entspannung.

*Anmerkung: Im BERN Manual steht die Faustübung im Kontext „Aktive Entspannung“, der mit dem Bild der „Wippe“ der Entspannungsantwort den dynamischen Prozess zwischen Anspannung und Entspannung aufzeigt (Tobias Esch, Sonja Maren Esch, 2016), p28*.

**Meditation-Entspannungsantwort (EA)**

#### Feedback Praxispartner

Ein TN hatte als „Meditationsobjekt“ das Wort „Ruhe“ ausgesucht – Hat sonst immer Angst, dass etwas nicht funktionieren könnte – „Wenn Sie meditieren, strahlen Sie eine große Ruhe aus.“ – „*Die Meditation ist eine Medizin ohne Medikamente*.“

Zwei der vier TN haben sich kein Wort, Geräusch oder sonstiges überlegt. Sie waren eher verwundert, als nachher danach gefragt wurde.

**Meditation im Tagesablauf - täglich (H) – (Meditation/Entspannungsantwort (EA) - täglich)**

#### Feedback Praxispartner

Die Meditation wurde ohne Anleitung nicht in den Tagesablauf integriert.

**Minis**

#### Feedback Praxispartner

Bei den Senior:innen des Praxispartners wurde die Verstetigung einzelner Meditationsübungen wie Zwerchfellatmung, usw. in einer Anleitung/Anleitungsvideo angeregt.

Aus den Feedbacks ergab sich, dass Zwerchfellatmung und Body Scan z.T. auch selbständig durchgeführt wurden, beispielsweise beim Einschlafen oder in Ruhezeiten, so gut es ohne „äußere“ Anleitung ging. – Gleichzeitig konnten sich die TN beim ersten Kennenlernen des Body Scan nicht gut vorstellen, die Übung alleine durchzuführen. Die Meditation/Entspannungsantwort wurde eher nicht selbständig in eine Verstetigung übernommen.

**Modul 3, Bewegung**

**Body scan**

#### Feedback Praxispartner

Der Body Scan wird sowohl beim ersten Durchführen als auch in der Wiederholung als sehr angenehm erlebt.

TN fühlen sich „sehr frei, positiv eingestellt. Keine negativen Gedanken“ besonders bei einem TN der „sonst immer Angst“ hat. „Man kann unheimlich viel mitnehmen …“

Eine TN hat alles verstanden, aber es ist im Kopf „so ganz langsam durchgegangen“. Sie ist gleichzeitig „richtig mitgelaufen“ – es war langsam genug und man ist richtig mitgekommen.

Alle TN haben sich über die „Ruhe“ der Übung gefreut, Begeisterung darüber, dass sie wirklich mit der Aufmerksamkeit genau an die Körperstelle folgen konnten, die in der Anleitung gerade „dran“ war. Als Positiv-Faktor wurde auch die ruhige Stimmlage benannt.

Es bestand – der Wunsch, diese Übung auf jeden Fall zu wiederholen.

Selbst den Body Scan durchzuführen wird eher nicht so angenommen, es besteht der Eindruck, dass man doch das Video braucht, - die Stimme … usw.

Es wird überlegt, den Body Scan zum täglichen Angebot zu machen.

**Yoga**

#### Feedback Praxispartner

Es gab gerade am Vortag eine Gymnastikstunde. Die TN haben daran gedacht … Die Anleitung aus unserem Übungsvideo wäre eine gute Möglichkeit für diese Gymnastikstunde.

Es gibt für zwei TN einen starken Bezug zum Thema Berg (Kontext: die Übung „Berghaltung“.) – Berge wurden in der Jugend erklommen, heute müssten die Berge ganz flach sein, damit man sie erklimmen kann. Man muss akzeptieren, dass was man im Leben noch schaffen kann, nicht mehr so ist, wie es mal war. Es besteht Angst, weil man die Dinge nicht mehr so schafft.

Man merkt gut, was man noch kann und was man besser nicht macht (lass das lieber sein, sonst fällst du hin). „Es gibt Tage wo es nicht so gut geht. Ich bin sehr vorsichtig geworden, gehe mit Rollator, habe Angst, wieder zu fallen.“

Es bestand generell Angst, hinzufallen; Orientierung im Raum schien schwer zu fallen und daher wurde die Übung nicht vollständig umgesetzt

(*Anmerkung: Hier wird deutlich, dass vor allem der Aspekt Bewegung, bzw. „Gymnastik“ erkannt wurde, jedoch der Zusammenhang „achtsames Bewegen“ eher nicht angekommen ist. -> In der Weiterentwicklung nach Möglichkeit berücksichtigen*.)

Die Dehnübungen fielen den Bewohnenden leichter. „Dabei kann man nachdenken über das, was vorher gesagt wurde … „

Übung Schultern nach vorne und nach hinten kreisen => Hierzu kam der Hinweis aus der Praxis: Nach Aussage des Sozialen Dienstes ist der unmittelbare Richtungswechsel erfahrungsgemäß ein Problem, denn der Richtungswechsel der anleitenden Person wird oft nicht gemacht bzw. nicht wahrgenommen, dass man tatsächlich wieder in dieselbe Richtung kreist … Alle bleiben dann beim Kreisen nach vorne. Dies ist kontraproduktiv für die ohnehin nach vorn zusammenfallende Haltung. Wenn man mit Kreisen nach hinten beginnt, wäre das Ende das Kreisen nach vorn, - ebenfalls kontraproduktiv. Daher eher auf das nach hinten Kreisen öfter hinweisen, … damit es wahrgenommen wird, eventuell die Arme seitlich strecken und eher „drehen“ lassen, dann wird der Richtungswechsel besser umgesetzt.

TN des Sozialen Dienstes: „Nach dem Ausschütteln hat es gekribbelt und sich lockerer angefühlt.“

Weitere TN: „Es wird helfen, wenn man die Übung mehrmals macht, dann weiß man immer schon was auf einen zukommt.“

„Es wäre schön, den Anstoß zu haben, die Übungen auch allein auf dem Zimmer zu machen, wenn man das Video verwenden kann. – Alleine ist man sonst zu faul – wenn man es vorgesetzt bekommt, macht man das eher …“

**Qi Gong**

#### Feedback Praxispartner (Meridiane)

Die Übung wurde als sehr angenehm empfunden. Teilnehmende waren sichtlich „happy“ nach der Übung. Alle konnten folgen und fanden auch, dass sie danach aktiver in den Tag gehen.

**Qi Gong- Reguliere den Atem**

#### Feedback Praxispartner (Reguliere den Atem…)

Die Dauer der Haltungen war sehr lang; Übungen wurden weitergemacht obwohl die Schmerzgrenze erreicht war; Feststellung: „Man will dann weitermachen … in der Gruppe … man ist ja ehrgeizig …“ - sehr anstrengend, hoher Ehrgeiz; Anleitung notwendig, um es mehrfach durchzuführen. (Es geht nicht mit einer Anleitung und dann der Aufforderung „wiederholen Sie die Übung mehrfach, wenn Sie mögen“).

Aus Sicht der Achtsamkeit ist das Ziel erreicht worden: es wurden persönliche Einstellungen und auch Ressourcen entdeckt, - wie „man“ so funktioniert … Ideal wäre es, diese nun in gesunde Bahnen zu führen, so dass die TN sich nicht übernehmen aber auch die Wahrnehmung in einer langsamen Körperübung nutzen, wenn sie bewusst an ihre Grenzen gehen. – Erklärungen erreichen die TN z.T. kognitiv nicht.

**Gehmeditation**

#### Feedback Praxispartner

Die Übung hat den Senior:innen nicht gefallen, war für sie nicht ansprechend; es war aus ihrer Sicht auch keine Meditation.

Zwei TN fühlten sich sehr wackelig und konnten nicht gehen. Eine TN hat ihren Rollator bekommen, ein TN hat sich am Stuhl festgehalten, ist aber nicht gelaufen. Insgesamt wäre es wohl besser, die Übung direkt mit Rollator anzuleiten. Ein TN hat sogar seine Schuhe ausgezogen. Er hat die Übung als Herausforderung beschrieben, die nicht entspannend war.

Die Nachfrage, ob die TN sich die Übung im Sitzen vorstellen könnten, ist nicht so richtig angenommen worden. Sitzgymnastik würden sie ja immer machen. Das bewusste Hineinspüren und bewusste Wahrnehmen von Schritten, war ihnen nicht zugänglich. Es wurde dann nochmals eine Erklärung dazu gegeben und eingeladen, gleich auf dem Weg aus dem Raum es nochmals auszuprobieren. – Kein weiteres Feedback zu dieser Übung erhalten.

**Rezept für Bewegung**

#### Feedback Praxispartner

Senior:innen wählten auf einem Vordruck für das Rezept für Bewegung bestimmte Übungen aus. Eine TN würde alle Übungen auswählen. Ein TN möchte sich ein Tablet anschaffen, wenn er von der Einrichtung Unterstützung bekommt, damit er die Übungen auch machen kann.

An sich wollen zwei der Senior:innen die Übungen lieber in der Gruppe durchführen. Ein Rezept an sich würden sie nicht benötigen.

**SARW (achtsam sein)**

#### Feedback Praxispartner

Die Übung wurde zuerst sprachlich verstanden, ist aber innerlich nicht angekommen. Alle TN empfinden. „Wenn ich das Signal Stopp bekomme, dann stoppe ich mit allem, auch mit den Gedanken. Nach dem Stopp kommt ein ganz neuer Gedanke …“ *(Anmerkung: Das ist ja im Sinne der Übung genau das Erwünschte.)–*

Am Anfang wurde die Frage gestellt „Will ich mir eine schwierige Situation überhaupt vorstellen?“ - Das „Stopp“ wurde als Befehl verstanden, als „du hast etwas falsch gemacht“ empfunden. “ - „Wenn das „Stopp“ kommt … wartet man auf den Atem … wann darf man wieder Atmen?“

Das Video ist so nicht handhabbar, für die Teilnehmenden „nicht lernbar“.

Interessanterweise wurde später, beim Fazit, von einem Senior mit einer demenziellen Veränderung ausgerechnet die SARW-Übung als die hilfreichste Übung von allen bewertet: „Stopp“ – das hole ihn auf dem Gedankenkreisen heraus. „Das Schönste war mit „Stopp“ – ich habe es beim Einschlafen geübt, wenn Gedanken kamen: „Stopp“.

**Modul 4, Ernährung**

**Achtsamkeitsübung Ernährung (Rosinenübung)**

#### Feedback Praxispartner

Alle Feedbackgebenden haben die Anleitung mitgemacht und konnten ihr folgen. Eine Person hat nichts besonders gemerkt. Der Geschmack war süßlich und säuerlich zugleich. Eine andere Person hat die Übung sehr genossen und sie den „intensiven“ Übungen zugeordnet. Er hat sich eine frische Weintraube vorgestellt. An seinem Haus hatte er zwei Weinreben. Er hat sich vorgestellt auf einem Weinberg zu sein. Er hat es knistern hören und auch den Speichelfluss bemerkt.

Zwei Teilnehmende fanden es ekelig die angefasste Rosine dann in den Mund zu nehmen.

Es ist die Idee entstanden, diese Übung auch mit anderem Essen zu machen: Tomate, Gurke, Erdbeere. Insgesamt erwarten wir eher nicht, dass diese Übung in den Alltag übernommen wird.

Auf Nachfrage zwei Wochen später: Manche Nahrungsmittel – wie bei der Rosinenübung – lösen bestimmte Vorstellungen aus (Weintraube, …) im Alltag wurde das noch nicht bewusst probiert. Nur eher bewertend: „das schmeckt mir“ oder „das esse ich gerne“. Wenn ich es nicht gerne esse, dann schmeckt es mir auch nicht. – Wir schmecken (hier in der Einrichtung) einen großen Unterschied zwischen „Fast Food“ (Catering Firma) und dem hier (von Mitarbeitenden) selbst Gekochten.

**Mediterrane Kost-> Achtsamkeitsübung Trinken**

#### Feedback Praxispartner

Das Beschreiben der Wahrnehmung beim Trinken fällt den Feedbackgebenden schwer; von Anleitenden wird eine Beschreibung (kalt und wabbelig) hineingegeben, damit konnte weitergearbeitet werden.

Man ist sich bewusst, dass man zu wenig trinkt. Gläser und Flaschen sind im Zimmer bereits aufgestellt. (*Tipp: Zettel aufhängen. – Nützt das dann noch?*)

Es kommen Fragen auf: Wie viel soll man trinken? Warum ist Flüssigkeit so wichtig? Warum spüren ältere Menschen keinen Durst?

Hier ggf. im Übungsmodul auch einen Hinweis auf Gesundheitsinformationen geben. => Frage an TE zu Inhalt und Umfang.

„Morgens die erste Tasse Kaffee, dann fängt der Tag gut an.“

„Wir haben Schwierigkeiten mit dem Trinken. Ich weiß, dass das nicht gut ist aber man vergisst es immer es dann.“

**20 Sachen -> 3 Sachen (Schatzkiste)**

#### Feedback Praxispartner

Der Blick auf das eigene Leben erzeugt auch negative Gedanken (Trennung von Frau; „abgeschoben“ worden;) aber auch: viele Dinge richtiggemacht

TN brauchen mehr Zeit, darüber nachzudenken, die Aufgabe hat sie überfallen.

Es sind auch intime Aspekte, die nicht in der Gruppe geteilt werden wollen.

**Achtsames Mahl**

#### Feedback Praxispartner

Bislang wurde nicht weiter darauf geachtet. TN eingeladen, dass sie mal auf den ersten Schluck Kaffee morgen achten oder den ersten Bissen der Waffeln ganz bewusst wahrnehmen.

Siehe Rosinenübung: Nur eher „Das schmeckt mir“ oder „das esse ich gerne“. Wenn ich es nicht gerne esse, dann schmeckt es mir auch nicht. – Wir schmecken einen großen Unterschied zwischen „Fast Food“ (Catering Firma) und dem hier (von Mitarbeitenden) selbst Gekochten.

**Geführte Imagination „Küche“**

#### Feedback Praxispartner

Starke emotionale Reaktion der TN auf die Vorstellung der eigenen Küche.

Aufgefangen durch: anschließende Zwerchfellatmung => wieder Ruhe ins System bringen.

Anregung: Nicht die eigene Küche sondern „eine Küche“ vorstellen? – Hier wäre das Ziel, die emotionale Belastung zu vermeiden.

*Vermutung: (1) Man wird trotzdem in der eigenen Küche „stehen“. Sie ist einem am nächsten und kommt in der Erinnerung zuerst. – (2) Außerdem wäre nochmal das Ziel der Übung zu klären: geht es wirklich darum, eine emotionale Belastung zu vermeiden? Die Intention könnte auch sein, zu sehen, wie sehr unsere Vorstellung unsere Gefühle und Gedanken beeinflusst und dass wir letzten Endes im Fahrersitz sitzen.*

**Modul 5, Soziales Netz, Freunde, Umfeld**

**Soziales Atom- Beziehungsgeflecht**

#### Feedback Praxispartner

Signal aus anderen reflektiven Übungen: TN wollten sich nicht mit „solchen“ Fragen befassen. Sie suchten eher Ablenkung.

Dennoch wurde diese Übung sehr gut angenommen. TN schreiben fleißig und folgen der Anleitung im Video, das zunächst bis 2:20 min gehört und dann pausiert wurde (später wurde das Video für den zweiten Übungsteil weiter abgespielt).

Sie seien im Alter eher festgefahren in den Interessen, sie würden nicht mehr so viel Neues angehen. Man habe so seine Lieblingssachen und ganz wichtig sei die Familie.

Die Idee des Sonnensystems finde sie gut und nachvollziehbar.

Die Übung hat dazu angeregt, nachzudenken „wie es mal weiter wird“. Auch das Nachdenken über die Nachkommen und wie die mal leben werden.

Die Übung hilft dabei, einem bewusst zu werden, was einem wichtig ist. Man wird daran erinnert, da mal bewusster drüber nachzudenken.

*(Hinweis: interessant ist, dass hier anscheinend keine negativen Emotionen ausgelöst wurden. Das Nachdenken „wie es mal weiter wird“ hört sich hier nicht so negativ an wie zuvor in einigen Übungen.)*

**Modul 6, Sprache und Ausdruck**

**Postkarte**

#### Feedback Praxispartner

TN haben die Übung Postkarte in der gemeinsamen Sitzung mit Wissenschaftlerinnen angesehen und als „Hausaufgabe“ für das nächste Treffen mitgenommen. Sie haben sich in Eigeninitiative am Tag vor dem gemeinsamen Termin getroffen, die Aufgabe gemeinsam besprochen, - übereinstimmend zu dem Ergebnis gekommen: Eine Postkarte, in der man sich selbst etwas Nettes schreibt, fühlt sich so an als würde man sich „in den Himmel Heben“, - dass „Eigenlob stinkt“.

Eine TN äußerte motorische Problem, eine Karte zu schreiben. Sie wollte es nicht machen, da es ihren Ansprüchen nicht genügt hätte.

„Schreiben packe ich nicht mehr so wie ich gerne möchte, - ich kann das so nicht abgeben. Ich muss, wenn ich etwas schreibe, mehrmals ansetzen. (Ergänzung auf Nachfragen: Gefällt inhaltlich nicht, auch das Schreiben an sich (Handhabung) fällt schwer.)“

Ein TN schrieb „die Gedanken sind frei und wir lassen sie laufen …“ und „ich kann es auch nicht schreiben. – Wenn ich grüße dann grüßt man ja auch nur im Positiven. Weil ich von meiner Frau verlassen wurde, prägt sich das so ein, dass kein anderer Gedanke Platz findet.“

Er las dennoch ein paar Zeilen vor, die er sich auf einem Papier notiert hatte. Er sagte, dass er nur negative Gedanken habe, da er von seiner Frau verlassen wurde.

Ein TN hat begonnen an sich zu schreiben. „es ist alles gut“ – „ich habe es aber nicht weggeschickt.“ Er hat einen Text an sich selbst verfasst, den er vorlas. Er hat sich nach dem Gespräch mit den anderen kollegial zurückgenommen und seine Postkarte dann auch nicht abgeschickt.

**Liebende- Güte Meditation**

#### Feedback Praxispartner

Bei einem TN hat es negative Gefühle ausgelöst; er fragt sich, wen er wirklich liebt. Er meint, manche Übungen würden sehr positiv wirken, manche bei ihm negativ. Er wird sich Frage mitnehmen, was ihm wirklich wichtig ist.

*Anmerkung: Was bedeutet negativ? – Heißt das, dass man auch über die schwierigen Seiten des Lebens nachdenkt? – Ist das wirklich „negativ“ bzw. muss es das sein? -Siehe oben: Diskussion zum Zweck der Übungen.*

Die anderen TN relativierten die Aussage. Sie meinten, dass die Übungen zum Nachdenken anregen.

**Modul 7, Selbsthilfe und Selbstheilung**

**Sinn und Zweck (Selbsthilfestrategien)**

#### Feedback Praxispartner

Die TN schrieben fleißig in der Pause der Anleitung (nach den Fragen: Sinn im Leben? Wofür lohnt es sich für mich zu leben?) und nach der Übung.

Reaktionen: Die Übung stimmt nachdenklich. Was kann man machen, um glücklich und zufrieden zu sein?´- Was will ich? Was will ich nicht? - Familie ist ein wichtiges Thema.

Dass die TN hier mit Papier und Bleistift geschrieben haben, scheint ein wichtiges und erfolgreiches Element der Übung zu sein. Dies evtl. auch auf andere Übungen ausweiten. Wir überlegen, auch begleitend zur App bei den relevanten Übungen das Notieren mit Papier und Bleistift anzuregen.

**Witze/Humor /Lachen**

#### Feedback Praxispartner

Das Lachen im Video war ansteckend; Corona-Regelungen erfordern Masken, die wiederum verhindern, dass ein Lächeln erkannt wird; das erzeugt eher depressive Stimmung (TN ist „an sich eine Frohnatur“).

**Modul 8, Rückfallprävention**

**Rezept-Gesundheitsfürsorge zur Selbstverordnung**

#### Feedback Praxispartner

Die Bewohner:innen hatten sich dieses Rezept ausgefüllt, es war ihnen zuvor ausgehändigt worden.

„Die Übung wurde auch als Hausaufgabe gemacht. Das Video dazu haben wir deshalb nicht angesehen.“

Ein TN hat alle Felder angekreuzt. Sozial „Das Wort Nein gibt es in meinem Vokabular nicht“ – ist aber auch ein Abgrenzungsthema.

Eine TN hat auch alles angekreuzt.

Ein TN hat bis zur Hälfte angekreuzt (nur die oberen Übungen). Er sagt: „Es muss auch möglich sein, „nein“ zu sagen.“

Ankreuzen ging auch leicht (leichter als selbst etwas zu schreiben).

Zitat Praxispartner: „Wir beschäftigen uns schon mit den Anregungen und Empfehlungen, die Sie (Projektteam UW/H) geben.“

**Fazit**

#### Feedback Praxispartner

TN sind durch die gemeinsamen Übungen auch als Gruppe zusammengewachsen. Auch eine TN, die zu Beginn sehr zurückhaltend war ist „aufgeblüht“ und erzählt in den Austausch-Runden viel.

B - Thema Verhalten … ja, es gibt Änderung – Mehr Gymnastik (Hr. Parton), aber das ist ganz anders. Gewohnheit – Man ist so beschäftigt und vergisst als was wir hier gemacht haben, „Die Sachen die wir hier gemacht haben könnte man auch hier im Hause gemeinsam üben. Manche Dinge sind so im Unterbewusstsein, Essen, Meditation, manchmal passt das zufällig und dann denkt man daran. Wenn die Situation da ist.“

Eine TN: Ganz sicher hat sich etwas verändert, ich kann jetzt nicht die Worte dafür sagen. – Wenn ich manchmal wach liege, denke ich „das könntest du jetzt machen“. Aber dann wird geklingelt oder es wird gewaschen, …

E - Thema Bewegung … wird 30 Minuten gemacht – oder wenn es der Körper erlaubt. Z.T. Spaziergang. – Übungen mit dem Oberkörper (wie in unseren Übungen angeboten) eher nicht.

R - Thema Entspannung … Entspannen kann man wenn man allein spazieren geht. Oder Familie und Besuch sind Entspannung. Oder aufs Bett legen und Body Scan üben. Kreuzworträtsel, Sudoku. Auch Aktivität entspannt. Zusammenkommen und etwas tun, was auch Spaß macht.

N - Thema Ernährung … ESSEN: Manche - wie Rosinenübung – lösen bestimmte Vorstellungen aus (Weintraube, …) im Alltag noch nicht bewusst probiert. Nur eher „Das schmeckt mir“ oder „das esse ich gerne“. Wenn ich es nicht gerne esse, dann schmeckt es mir auch nicht. – Wir schmecken einen großen Unterschied zwischen „Fast Food“ (Catering Firma) und dem hier (von Mitarbeitenden) selbst Gekochten.

TRINKEN: morgens die erste Tasse Kaffee, dann fängt der Tag gut an.

Wir haben Schwierigkeiten mit dem Trinken. Ich weiß, dass das nicht gut ist aber man vergisst es immer.

Kein Mensch ist vollkommen …

Lieblingsübung eines TN: Body Scan

Lieblingsübung eines TN: „Stopp“ – das hole ihn auf dem Gedankenkreisen heraus: Das Schönste war mit „Stopp“ - beim Einschlafen geübt, wenn Gedanken kamen „Stopp“. Auch die Übung mit dem Körper, Body Scan.

Vielen Dank für die Teilnahme! Sie dürfen die Übungen (Links wurden dem Sozialen Dienst übermittelt) sehr gerne weiterverwenden.
