# Supplementary material for: Development of a (digital) mindfulness-informed intervention for older adults in nursing homes: description and reflection of a person-based co-design approach
Source: BMC Geriatr. 2025 Sep 25;25:703. doi: 10.1186/s12877-025-06223-x (PMC12462116; doi:10.1186/s12877-025-06223-x)
Supplement: Supplementary file 2 — Supplementary Material 2. [file 12877_2025_6223_MOESM2_ESM.docx]

Appendix 2: Online Survey with stakeholders

Table: Feedback on exercise and implementation of exercise

|  | Choice Statements | Action required if selected |
| --- | --- | --- |
| Feedback on Exercise | The exercise is suitable for the target group without any adjustments | No action required |
|  | The exercise is only suitable for the target group with adjustments | The qualitative feedback may indicate necessary adjustments. Adjustments within the framework of the model |
|  | The exercise is not suitable for the target group | Check whether the exercise meets the project requirements and collect the feedback |
|  | The residents of a nursing facility will get involved in the exercise | No action required |
|  | “Other” (option to enter free-text input) | Open answer |

| Feedback on implementation of exercise | Participants can follow the exercise | No action required |
| --- | --- | --- |
|  | Participants can rather not follow the exercise | Determine the cause if selected by relevant number/majority; The qualitative feedback may provide some guidance. Adjustments within the framework of the model |
|  | The explanation needs some improvement | Improvement of the explanation of the exercise |
|  | Participants need more instructions | More guidance during the exercise |
|  | The exercise requires (too) demanding cognitive abilities | Make exercise easier |
|  | “Other” (option to enter free-text input) | Open answer |

The open answers correspond to the following questions posed to the survey participants:

- If you checked the answer option “The exercise is only suitable for the target group with adjustments” for the exercise “[name of exercise]”: What adjustments do you suggest?
- Which advantages do you see in using this exercise?
- Which difficulties do you see in using this exercise?
- How would you address these difficulties?
