# Supplementary material for: Development of a (digital) mindfulness-informed intervention for older adults in nursing homes: description and reflection of a person-based co-design approach
Source: BMC Geriatr. 2025 Sep 25;25:703. doi: 10.1186/s12877-025-06223-x (PMC12462116; doi:10.1186/s12877-025-06223-x)
Supplement: Supplementary file 4 — Supplementary Material 4. [file 12877_2025_6223_MOESM4_ESM.docx]

# Appendix 3: Category system (definitions and anchor examples)

| **Subcategory** | **Definition** | **Anchor example(s)** |
| --- | --- | --- |
| **Formulation** | | |
| Lighter/unclear wording | Articles that address the understanding of specific formulations are assigned to this subcategory. This includes, among other things, sentences that are incomprehensible due to their length, but also the preferred type of sentence (more questions) and suggestions for rephrasing. | “[ …]“ What would you like to achieve that would, for example, give you relaxation or pain relief?” Wouldn’t relaxation or pain relief be a goal? Otherwise I don’t really understand the sentence.”  "Are you still breathing?" I would change it to "Are you still breathing deeply?" (2:30) |
| Other name | Articles that criticize the use of specific terms are assigned to this subcategory. There is little discriminatory power for the first subcategory | “Perhaps new OR good would be a better fit?”  “The term “sources of strength” is not necessarily common, nor is it that easy to adapt to the target group. Maybe something like “courage to live”/“strength” would also fit.” |
| Including wording | Contributions that suggest a change in terms of the inclusion of all participants are assigned to this subcategory. This includes, in particular, naming aids and offering assistance from staff. | “For wheelchair users, maybe just say “sit comfortably” and not necessarily “in a chair”?”  “Can the residents still write? Isn't it disheartening when they can no longer do that? Maybe add “write it down or have it written down” |
| Other | Related contributions related to the formulation are assigned to the subcategory Other. |  |
| **Content** | | |
| Explain the term/added value | This subcategory includes contributions that indicate that a procedure or term requires further explanation. A crucial difference to the formulation category is that a new text module is necessary and a reformulation is not enough. | “[ …]Is there an explanation somewhere as to what meridians actually are? Then perhaps the meaning of the exercise would be better understood.”  “In the introduction about “stress mechanisms” either leave out the speech or explain what is actually happening here and what connection the fist/tension has with the stress .” |
| Give examples | suggest increased comprehensibility by including additional examples are assigned to this subcategory | “Example mentions of “treasures” are missing.”  “In order to understand the “social atom” and transfer it to yourself, explanations are required that include your living environment.” |
| Other | Adjacent posts related to the content are assigned to the Other subcategory. |  |
| **Execution** | | |
| Series | This subcategory includes contributions that either criticize the positioning of an exercise within the entire intervention or the order within an exercise. | “Not as a first exercise, first get to know a tool for dealing with stress.”  “Either leave out the hint that you should finish when it suits you and direct your return directly with the hint that the exercise will now end, or a hint that if you want to stay in it longer, you can stop the video now and again turns on when you want to end the exercise. But I personally find the latter rather unsuitable.” |
| Gong | This subcategory includes contributions that (directly or indirectly) address the gong. | “In the course of the aging process, older and very old people lose the ability to hear high-pitched sounds. Maybe you can choose a slightly deeper "gong" - I personally would find it a little more contemplative and calmer."  “I would create a clearer distinction between background information and the start of the exercise” |
| Other | Related contributions relating to implementation are assigned to the subcategory Other. |  |
| **Presentation** | | |
| Tempo | This subcategory includes contributions that criticize the pace of the speakers (as a whole or at individual points). This often affects breaks. | “A little slower might be even better”  “A slightly longer break after “take a moment ” would be quite good” |
| Emphasis | Contributions that address the connotation or style of the person presenting are assigned to this subcategory. | “Overall, the exercises could be performed with a little more pepper and more lively”  “S. My note about the tone of voice, temperament in the description , etc. for the first videos. The soporific tone and the lack of gestures don’t exactly make you want to try out what’s being said.” |
| Body language | Contributions that highlight the facial expressions and/or gestures of the speaker are assigned to this subcategory. | “You can simply make the gesture for “hold your heart” larger and show the upper body sufficiently so that it becomes clear or leave the gesture out.”  “I would like to smile more” |
| Image detail | Articles that relate to the selected image section in the preliminary video recordings are assigned to this subcategory. | “The actors should be presented in more detail […]”  “If you want the gestures to come across, I would make sure that the upper body is clearly visible and that you choose big movements.” |
| Other | Related posts related to the presentation are assigned to the Miscellaneous subcategory. |  |
| **Technology** | | |
| (Sound) quality | Articles that criticize the technical quality of the videos are assigned to this subcategory. | “The sound quality should be a little better”  “Sound quality is not good - too much background noise and too reverberant ” |
| Technical introduction | This subcategory contains contributions that address the need for a technical introduction to the participants. There is little discriminatory clarity regarding the general topic of accompaniment. | “Do the residents already know about the app or how to get in here?”  “I don’t quite understand the last comment “If you want, you can speak or write your source of strength into our app in the future” – how is this specifically addressed and explained? In workshops or can they then address nursing staff or what would the process be? – This question also applies to the other videos” |
| Other | Related articles relating to technology are assigned to the subcategory Other. |  |
| **Accompaniment** | | |
| Inefficient implementation without supervision | This subcategory includes contributions that represent the view that the exercise can only be carried out for the target group if support (e.g. nurses, trainers ) is available. | “Meditation exercises are unfamiliar to the target group. I doubt she will commit to this without individual support. The majority of those addressed will not understand the meaning and will react with rejection and fear.”  “Accompany residents during the task and support them if necessary.” |
| Accompanying conversations | Articles that suggest accompanying discussions are assigned to this subcategory. This can include both preliminary and debriefings. | “ I would also like a reflection session , as long as it takes place in a group”  “Starting up in the follow-up conversation” |
| Other | Related contributions relating to accompaniment are assigned to the Other subcategory. |  |
| **Feasibility concerns** | | |
| Cognitive concerns | This subcategory includes contributions that express concerns of a cognitive nature. The concerns relate to the feasibility of the exercise in the target group. | only reason I didn't tick the above answer option "The exercise requires too high cognitive abilities " is because, in relation to our target group, I would rather call it "The exercise requires high cognitive abilities." Since some of the facility's group offerings take place with mixed "target groups" , it is possible that some would be overwhelmed, while others are still able to absorb what is being said. Therefore, I would only use this exercise if I could inspire interested residents to get involved in it.”  “Difficulties are to be expected in the area of concentration. The exercise requires a high level of concentration, which may not always be present or could be quickly interrupted.” |
| Emotional concerns | Posts that express concerns of an emotional nature are assigned to this subcategory. The concerns relate to the feasibility of the exercise in the target group. | “The undesirable side effect of making single people feel bad is just an eventuality.”  “If you think about it, some residents might feel sad. Home as the “final stop”.” |
| Physical concerns | Posts that express concerns of a physical nature are assigned to this subcategory. The concerns relate to the feasibility of the exercise in the target group. | “some people may not be able to walk overhead, this could pose a difficulty”  “People with balance issues could potentially have trouble or fall while doing this exercise.” |
| Other | Related contributions related to feasibility concerns are assigned to the Other subcategory. |  |
