# Supplementary material for: Development of a (digital) mindfulness-informed intervention for older adults in nursing homes: description and reflection of a person-based co-design approach
Source: BMC Geriatr. 2025 Sep 25;25:703. doi: 10.1186/s12877-025-06223-x (PMC12462116; doi:10.1186/s12877-025-06223-x)
Supplement: Supplementary file 5 — Supplementary Material 5. [file 12877_2025_6223_MOESM5_ESM.docx]

**Modul 1 Stress und Stressbewältigung**

**Ziele setzen**

#### Anpassungen

- Es wird in dieser Übung kein Gong verwendet.
- Es wurde ein weiteres Beispiel und zusätzliche Fragen zur Orientierung, zur Bildung von Assoziationen zur Beschreibung des Konzeptes „Ziel“ eingefügt
- Hinweis, dass beim Notieren oder Einsprechen des Ziels Hilfe (von begleitender Person) in Anspruch genommen werden kann.

**Kraftquelle**

#### Anpassungen

- Beispiele hinzugefügt: Auch die Erinnerung an liebe Menschen oder ein Andenken kann eine Kraftquelle sein
- Zusätzliche Fragen eingefügt, die das Konzept Kraftquelle verdeutlichen Hinweis, dass beim Notieren oder Einsprechen des Ziels Hilfe (von begleitender Person) in Anspruch genommen werden kann.

**Neues und Gutes**

#### Anpassungen

- Ermunterung zur regelmäßigen Übung, Motivation
- Vorschlag Hilfsmittel (Notizbuch)
- Hinweis, dass beim Notieren oder Einsprechen des Neuen und Guten Hilfe (von begleitender Person) in Anspruch genommen werden kann.

**Modul 2, Entspannung**

**Zwerchfellatmung**

#### Anpassungen

- Reihenfolge: Zwerchfellatmung vor Faustübung einführen
- „Fokussieren“ ersetzt durch „wahrnehmen“
- Beschreibung der Atmung vereinfacht
- Anweisung „tief“ einatmen ersetzt durch Beschreibung mit Bezug auf die individuellen Möglichkeiten
- Atemzüge zählen klar als optional hervorgehoben
- Ermutigung zum Üben ergänzt

**Faustübung**

#### Anpassungen

- Hinweis ergänzt, dass die Übung auch auf Vorstellungsebene durchgeführt werden kann, beispielsweise bei körperlichen Einschränkungen.
- Einleitung von der Übung absetzen, Gong zu Beginn der eigentlichen Übung.
- Bezug „Stuhl“ entfernt. -> Sitzen Sie bequem. Bezug auf Sitzfläche.

**Meditation- Entspannungsantwort**

#### Anpassungen

- Das Wort „Fokus“ gestrichen
- Sitzfläche statt Stuhl
- „Konzentrieren“ ersetzt durch „richten Sie Ihre Aufmerksamkeit“
- Beispiele für Geräusche, Bilder ergänzt
- Mehr Raum für individuelle Ausgestaltung
- Konkrete Zeitbenennung (5-10 Minuten) ersetzt durch „einige Minuten“
- Individuelles Beenden wird in der Anleitung deutlicher gemacht
- Das Video bleibt ungekürzt, es werden aber zwei Längen angeboten (ca. 5 und ca. 10 Minuten)

Diese Übung ist ein Kernelement des BERN-Konzepts und soll daher auf jeden Fall in den Interventionsgruppen umgesetzt werden.

**Meditation im Tagesablauf**

#### Anpassungen

- Die Beschreibung der Meditation als tägliche Übung soll im Kursablauf nach dem Body Scan und der Gehmeditation kommen, da diese am Ende als besonders geeignete Übungen verlinkt werden, oder es soll in geeigneter Weise hingewiesen werden, dass diese beiden erst in Woche 3 geübt werden.
- Für die Entspannungsantwort besonders geeignete Übungen werden unter dem Anleitungsvideo verlinkt
  - Body Scan
  - Gehmeditation
  - Zwerchfellatmung
- Übungstagebuch ist optional, Notizblock wird bereitgestellt

**Minis**

#### Anpassungen

- Als Minis besonders geeignete Übungen werden unter dem Anleitungsvideo verlinkt
  - Zwerchfellatmung
  - Meditation
  - Faustübung
- Noch deutlichere Anregung zum selbst-Üben

**Modul 3, Bewegung**

**Body Scan**

#### Anpassungen

- Ca. 10-14 Minuten in der vorliegenden Fassung.
- Zusätzlich eine längere Version anbieten => Raum zum Wahrnehmen länger, evtl. Ausgestaltung durch Sprecherin
- Organe wahrnehmen vielleicht nur in der längeren Fassung?

**Yoga**

#### Anpassungen

- Ergänzung: Thema „achtsames Bewegen“ wird im Anleitungstext aufgegriffen. Auf das Thema „Eigene Grenzen wahrnehmen“ wird ebenfalls eingegangen.
- Evtl. Schulterprobleme zu erwarten => stärkere Berücksichtigung möglicher körperlicher Einschränkungen in der Anleitung
- Pause zwischen Richtungswechseln einfügen und die neue Richtung explizit anleiten (sh. Feedback Praxispartner)
- Deutlichere Aufteilung der einzelnen Übungen

**Qi Gong**

#### Anpassungen (Meridiane)

- Begriff Meridiane erklärt
- Abstreichen statt abklopfen als Alternative beschrieben

**Qi Gong – Reguliere den Atem**

#### Anpassungen (Reguliere den Atem…)

- Hinweis: eigene Grenzen beachten.
- Auf der Vorstellungsebene ausführen, wenn man die Übung wegen Einschränkungen nicht ausführen kann

**Gehmeditation**

#### Anpassungen

- Hinweis auf die Verwendung von Hilfsmitteln ergänzt (Gehstock, Rollator)
- Darstellung des Gehens mit Rollator
- Hinweis: die Übung kann auch im Geist gemacht werden
- Abrollenden Fuß darstellen

**Rezept für Bewegung**

#### Anpassungen

- Erinnerungshilfe durch Postkarte (war bereits geplant)
- Bewegungsübungen werden unterhalb des Videos verlinkt

**SARW achtsam sein**

#### Anpassungen

- Information über die erfolgte Übungsanpassung wurde aus der Anleitung herausgenommen
- Begriff „Bewusstheit“ statt „Kontrolle“ verwendet
- Gong anders gesetzt, Erklärung besser abgegrenzt
- Kognitive Ansprache/Erklärung ergänzt

**Modul 4, Ernährung**

**Achtsamkeitsübung Ernährung (Rosinenübung)**

#### Anpassungen

- Frucht in die Hand nehmen -> darstellen
- Rosine als eine Möglichkeit, weitere Alternative aufzeigen („andere getrocknete Früchte“)
- Körperliche Einschränkungen/Allergien, die am Durchführen der Übung hindern könnten, im Vorfeld ansprechen

**Mediterrane Kost -> Achtsamkeitsübung Trinken**

#### Anpassungen

- Erinnerungshilfe
- Kleinere Anpassung im Text
- „Etwas zu Trinken“ wird bei den Hilfsmitteln zur Übung benannt
- Personen mit einer Herz- oder Niereninsuffizienz könnten Restriktionen bei der Trinkmenge haben, dies wird im einleitenden Text erwähnt. (*Hinweis: In der Studie: Berücksichtigung bei Auswahl der Teilnehmenden. Außerhalb der Studie: durch Pflegepersonen abklären ob/in wieweit eine Teilnahme an dieser Übung möglich ist.*)

**20 Sachen -> 3 Sachen (Schatzkiste)**

#### Anpassungen

- Erinnerungshilfe (Post It?)
- Kleinere Formulierungen angepasst
- Hinweis auf Schatzkiste „in der Vorstellung“ deutlicher erklären

**Geführte Imagination „Küche“**

#### Anpassungen

- Kleinere Anpassungen im Text (beispielsweise „die Küche“ statt „Ihre Küche“, „Vorstellung“ statt „Imagination“)
- Stuhl nicht benannt, stattdessen „Sitzfläche“
- Ergänzung: „Es gibt bei dieser Übung nichts zu erreichen oder festzuhalten.“ Nehmen Sie sich Zeit, Ihre Empfindungen wahrzunehmen“, mit Pause zum Wahrnehmen.

**Modul 5, Soziales Netz, Freunde, Umfeld**

**Soziales Atom- Beziehungsgeflecht**

#### Anpassungen

- Weiteres Beispiel (Pflegepersonal) eingefügt
- Pausen zum Einzeichnen und Nachdenken eingefügt
- „Persönliches Universum“ angesprochen
- Darstellung der Planeten/-bahnen im Video

**Modul 6, Sprache und Ausdruck**

**Postkarte**

#### Anpassungen

- Input zum Thema Selbstannahme / Selbstfürsorge ergänzt. „Seien Sie einmal für sich selbst ein ‚bester Freund‘ oder eine ‚beste Freundin‘!“
- Beispiele ergänzt
- Ergänzt: Möglichkeit, ein Bild zu malen statt Worte zu schreiben
- Hilfe bei Bedarf in Anspruch nehmen

**Liebende- Güte Meditation**

#### Anpassungen

- Kleinere Anpassungen im Text (Sitzfläche statt Stuhl,
- Statt „frei von Leiden“ -> „in Frieden sein“)
- Infinitiv statt Imperativ
- „Wir“ an einigen Stellen gestrichen

**Modul 7, Selbsthilfe und Selbstheilung**

**Sinn und Zweck (Selbsthilfestrategien)**

#### Anpassungen

- Pausen eingefügt
- Formulierungen vereinfacht

**Witze/ Humor/ Lachen**

#### Anpassungen

- Keine geplanten inhaltlichen Anpassungen
- Anpassung in der technischen Umsetzung: mit Gesichtern lachender Menschen, statt mit Video des Lach-Flashmobs; ersetzt aus Copyright Gründen.

**Modul 8, Rückfallprävention**

**Rezept- Gesundheitsfürsorge zur Selbstverordnung**

#### Anpassungen

- Benennung der Übung vereinheitlicht
- Markierung der Lieblingsübungen beschrieben

**Fazit**

#### Anpassungen

- Kleinere Anpassungen in Formulierungen
- Link zur Übungsübersicht bereitstellen (Frage: Wie kommen die TN wieder zurück?)
